# Biosecurity implementation in indoor and outdoor-access broiler production systems in the Netherlands

**DOI:** 10.1016/j.psj.2026.107951

**Published:** 2026-10-12

**Authors:** Yasmin Bakhshi, Egil A.J. Fischer, Jan van den Broek, J. Arjan Stegeman, Francisca C. Velkers

**Affiliations:** Department of Population Health Sciences, Faculty of Veterinary Medicine, Utrecht University, Yalelaan 7, Utrecht, 3584 CL, the Netherlands

**Keywords:** Biosecurity, Broiler, Avian influenza, Production system, Biocheck.UGent

## Abstract

Good biosecurity practices on poultry farms can improve animal health, welfare, and productivity, but implementation varies considerably between farms. Recurrent highly pathogenic avian influenza (HPAI) outbreaks and the expansion of higher-welfare systems with outdoor access have raised concerns about potential differences in exposure risk. However, it remains unclear to what extent biosecurity implementation differs between production systems. A cross-sectional comparative study was conducted to assess HPAI-related biosecurity practices and risk factors in Dutch broiler farms, comparing intensive systems (indoor-only) and extensive systems (slower-growing broilers with covered outdoor access). The study included 78 farms (63 intensive and 15 extensive). Biosecurity was assessed using a harmonized subset of the Biocheck.UGent survey. From 40 comparable variables, 28 were selected based on relevance to HPAI introduction and transmission pathways. Differences between production systems were evaluated using Bayesian logistic regression with horseshoe priors. Most biosecurity measures did not differ substantially between systems, with considerable variation observed within both intensive and extensive farms. The largest estimated differences involved carcass storage cleaning, stocking density, and chick delivery frequency, although the statistical support for these differences varied. Stocking density and delivery frequency reflected structural differences between systems, whereas carcass storage cleaning reflected differences in management practices. These findings suggest that interventions targeting farm-specific weaknesses, supported by tailored biosecurity plans and on-site coaching, are likely to be more effective than system-based recommendations alone.

## Introduction

Highly pathogenic avian influenza **(HPAI)** remains a persistent threat to the poultry industry, with outbreaks causing substantial economic losses and raising public health concerns worldwide (Xie et al., 2023; Fusaro et al., 2024). The Netherlands, with its high density of poultry farms and water-rich areas that attract migratory and resident waterbirds, has experienced repeated HPAI introductions, resulting in large-scale culling operations and movement restrictions (Schreuder et al., 2022). Across Europe, poultry production is vulnerable to HPAI due to the concentration of farms, frequent animal and vehicle movements, and exposure to wild waterfowl along major flyways (Hautefeuille et al., 2020; Cullen et al., 2025; EFSA, 2025b).

Recent HPAI seasons have affected a broad range of commercial poultry categories across the European Union **(EU)**, including the Netherlands, with outbreaks recorded across both indoor and outdoor production systems (EFSA, 2025a, b). This vulnerability extends to broiler production, particularly as some systems incorporate outdoor access for animal welfare, which has been associated with a higher risk of HPAI introduction (EFSA AHAW Panel, 2017). Outbreaks in both indoor and outdoor systems raise questions about whether biosecurity practices differ systematically between them.

Biosecurity measures are the primary means of reducing the risk of HPAI introduction and subsequent within- and between-farm transmission (EFSA AHAW Panel, 2017), encompassing both external biosecurity aimed at preventing pathogen entry and internal biosecurity aimed at limiting spread once introduced (Dewulf and Van Immerseel, 2018). Key pathways for HPAI introduction into broiler flocks include contaminated vehicles and equipment, personnel movements, and direct or indirect contact with wild birds, all of which are primarily targeted through external biosecurity measures (EFSA AHAW Panel, 2017; Guinat et al., 2020). Because multiple introduction pathways coexist, effective mitigation requires consistent biosecurity implementation; however, implementation and compliance vary considerably across farms (Tilli et al., 2022; Vougat Ngom et al., 2025; Castillo et al., 2026).

Broiler production in Europe encompasses a range of systems, commonly categorized as intensive or extensive based on stocking density, breed type, and outdoor access. Intensive systems typically use fast-growing breeds with production cycles of 35 to 42 days and stocking densities up to or exceeding 33 kg/m², as permitted under EU regulations (Council of the European Union, 2007). In contrast, extensive systems use slower-growing breeds with longer production cycles (56 to 81 days), lower stocking densities, and often provide outdoor access (European Commission, 2008). These structural differences are expected to influence exposure pathways differently (Gonzales et al., 2013), with intensive systems characterized by more frequent vehicle and personnel movements due to shorter production cycles, and extensive systems by increased contact with wild birds and environmental contamination through outdoor access (EFSA AHAW Panel, 2017; Fusaro et al., 2024). Consistent with this, the rate of introduction of low pathogenic avian influenza has been shown to differ between Dutch poultry production sectors (Gonzales et al., 2013).

Current biosecurity research has focused primarily on conventional systems, with limited assessment of how production system is associated with biosecurity requirements and HPAI risk (Vougat Ngom et al., 2025). Moreover, inconsistent definitions of “intensive” and “extensive” systems limit cross-study comparisons (Vougat Ngom et al., 2025). Although some studies have compared biosecurity practices across countries, regions, or poultry types, direct comparisons between intensive and extensive broiler systems using harmonized, HPAI-relevant measures are scarce (Van Limbergen et al., 2018; Delpont et al., 2021; Mallioris et al., 2023). Furthermore, substantial variation in biosecurity practices exists between farms within the same production system, suggesting that farm-specific characteristics and local context are important determinants of implementation (Guinat et al., 2020; Islam et al., 2024). Practical constraints, including time, cost, structural limitations, and farm layout, have been identified as key barriers to compliance (Amalraj et al., 2024; Souillard et al., 2024), whereas farmers in extensive or organic systems may perceive certain measures as unsuitable, leading to selective implementation (Castillo et al., 2026). Together, these findings raise the question whether variation in biosecurity performance is aligned with production system or distributed across individual farms.

In the Netherlands, both conventional indoor-only broiler production and alternative concepts with slower-growing broilers and outdoor access are widely present (Slegers et al., 2024), so the 2 systems can be compared under the same national regulatory context. The Biocheck.UGent survey provides a standardized tool for assessing biosecurity across different production systems, enabling direct and consistent comparison (Mallioris et al., 2023). To inform more targeted prevention strategies, this study compared HPAI-related biosecurity practices between intensive and extensive Dutch broiler farms using a harmonized subset of Biocheck.UGent questions aligned with recognized pathways of HPAI introduction and spread. Because the number of biosecurity variables was large relative to the number of farms, differences between systems were estimated using Bayesian logistic regression with a regularized horseshoe prior, which shrinks estimates for variables with little support toward zero while retaining stronger signals in the data (Piironen and Vehtari, 2017). We hypothesized that although system-level structural characteristics would differ, substantial variation in biosecurity practices would remain between farms within each system.

## Materials and methods

### Study design and farm selection

A cross-sectional study was conducted to compare biosecurity practices and risk factors between intensive and extensive broiler farms in the Netherlands. The study included 78 Dutch broiler farms, comprising 63 intensive farms surveyed between 2015 and 2023 and 15 extensive farms visited between December 2023 and May 2024. This study was reported in accordance with the Strengthening the Reporting of Observational Studies in Epidemiology **(STROBE)** guidelines for cross-sectional studies, and the completed checklist is provided in Supplementary Material G.

For the purpose of this study, production systems were operationally defined based on outdoor access, with indoor-only systems classified as intensive and systems providing outdoor access classified as extensive. The extensive system included farms certified under welfare schemes such as “Better Life” (Beter Leven), a Dutch animal welfare certification program introduced by the Dutch Society for the Protection of Animals, characterized by higher-welfare standards, including slower-growing breeds, lower stocking densities, daylight in poultry houses, and covered outdoor runs. Farms were excluded if they were in a transitional phase, e.g., holding “Better Life” certification but without yet implementing a covered outdoor range, or operating specialized poultry housing systems targeting niche markets (e.g., Kipster), as these are not representative of broader industry practices.

The 63 intensive farms were obtained from the Biocheck.UGent database, which contains previously completed surveys from Dutch broiler farms. Because extensive broiler farms were not represented in this database, the 15 extensive farms were recruited separately through professional networks within the Dutch poultry industry, including veterinary contacts and industry associations. Recruitment was facilitated by veterinarians who approached farmers within their practice; the total number of farmers approached was not recorded, precluding assessment of participation rate. Of those who agreed to participate, a small number were excluded based on the exclusion criteria defined above (i.e., transitional certification status or a niche production system). No formal sample size calculation was conducted, as the parameters required for such a calculation (the anticipated effect size and its variability) were not available at the time of study design. For the extensive group, all eligible farms that consented to participate were included. For the intensive group, the sample comprised all Dutch broiler farm surveys available in the Biocheck.UGent database at the time of data extraction (17 May 2024). The 63 intensive surveys represent approximately 10% of the 613 broiler farms operating in the Netherlands in 2023 (CBS, 2025). The Biocheck.UGent intensive broiler survey does not record flock size and records location only at province level.

### Biosecurity assessment and data collection

Biosecurity was assessed using the Biocheck.UGent scoring system, a validated, risk-based tool that systematically evaluates on-farm biosecurity in poultry production (Van Limbergen et al., 2018; Caekebeke et al., 2020). Biocheck.UGent quantifies on-farm biosecurity across weighted domains and has been widely adopted across EU and non-EU countries (Mahmood et al., 2026). Two survey versions were used: the extensive broiler survey (100 questions) and the intensive broiler survey (79 questions). Both covered topics including infrastructure and location, farm organization, visitors and personnel, broiler movements (chick purchase and broiler removal), feed and water management, waste and carcass management, disease management, and cleaning and disinfection. Each question offered multiple response options, which were numerically coded, with category 1 representing the reference category (best biosecurity practice) and higher numbers indicating either greater environmental exposure (e.g., proximity to water bodies) or lower implementation of recommended biosecurity practices.

Data for extensive farms were collected through in-person farm visits between 7 December 2023 and 2 May 2024. Surveys were administered by researchers or veterinarians who directly observed biosecurity practices and completed the questionnaire on-site, thereby reducing potential reporting bias. To promote consistency, all assessors agreed beforehand on the interpretation of the survey questions and on what to observe, and visits not conducted by the main assessors were documented with photographs and written descriptions of the observations. Farmers provided written informed consent prior to participation. Data for intensive farms were obtained from the Biocheck.UGent database (survey years: 2015 to 2023), with exclusive access to raw data granted for this study. Surveys were completed under a range of user accounts, including farmers and researchers; however, the specific method of data collection for each submission could not be retrospectively determined. Consent for data use had been obtained at the time of completing the survey.

### Data cleaning and harmonization

To enable comparison between production systems, the 2 survey versions were harmonized. Two researchers with veterinary and biosecurity expertise independently reviewed both surveys to identify analogous questions and reconcile differences, standardizing responses to maintain comparability. Discrepancies were resolved through discussion until consensus was reached. Full harmonization mapping, composite variable construction, and final response coding are documented in Supplementary Material A. Where questions addressed similar biosecurity practices but offered different response options, categories were aligned and recoded to a consistent ordinal scale, with category 1 representing lowest risk. This ensured comparability and preserved the relative ordering of biosecurity risk across response options. Interdependent questions were combined into composite variables where appropriate to capture the intended construct. Where merging was not feasible, response categories were standardized across surveys.

Ten questions were excluded prior to harmonization based on 2 criteria (see Supplementary Material A for the complete list of excluded questions). First, 9 questions were removed due to low variability. Because the extensive group included only 15 farms, low variability was defined as at least 14 of 15 extensive farms falling into a single response category, leaving at most 1 farm across all remaining categories, too few for stable estimation. The equivalent proportion was applied to the intensive farms, corresponding to at least 59 of 63 farms. A question was removed only when the low-variability threshold was met in both production systems, so variables with little variation in only 1 production system were retained. Second, 1 additional question was excluded due to missing data, with 33 of 63 responses (52.4%) absent in the intensive farm dataset, precluding reliable between-system comparison. Apart from this question, none of the 40 harmonized variables had missing responses; all 63 intensive surveys were complete, and none was flagged as a test entry in the database. The 2 question-exclusion criteria (low variability and high missingness) were predefined a priori, before any examination of system-level differences, to avoid data-driven variable selection. This ensured the analysis focused on variables with sufficient variation in at least 1 production system. No other missing data were present. Where response categories had very low cell counts, adjacent categories were merged prior to modeling to ensure model stability (see Supplementary Material A). After harmonization, 40 questions were retained as comparable across both datasets; the 2 questions that differed in framing are described in Supplementary Material A. For each variable, category 1 (best biosecurity practice, lowest risk) served as the reference category in the Bayesian regression model.

The same 2 researchers independently assessed the 40 harmonized questions for relevance to HPAI-related biosecurity risks. The assessment was informed by published evidence on HPAI introduction and transmission pathways, including major reviews, evidence syntheses, and epidemiological studies addressing wild bird exposure, farm biosecurity, and direct and indirect between-farm contacts (Ssematimba et al., 2013; EFSA AHAW Panel, 2017; Guinat et al., 2020; Hautefeuille et al., 2020; Verhagen et al., 2021). Questions were considered relevant if they addressed a pathway with documented evidence of HPAI introduction or spread, including contact with wild birds and other animals, contaminated vehicles and equipment, personnel and visitor movements, and manure or carcass management, or a farm characteristic plausibly related to HPAI exposure, transmission, or implementation of biosecurity measures. Discrepancies were discussed and resolved by consensus. Based on this assessment, 28 questions were selected from the full set of 40 (see Supplementary Material A for the complete list of all 40 variables, codes, and response categories).

### Statistical analysis

All analyses were performed in R (version 4.5.2; R Foundation for Statistical Computing, Vienna, Austria). Descriptive statistics (frequency distributions and percentages) were computed for all 40 harmonized variables, stratified by production system (see Supplementary Material B). These descriptive results are reported alongside model outputs to aid interpretation of observed system-level associations. For visualization, risk scores were rescaled to a 0 to 1 range by min–max normalization, with the lowest response category (best practice) set to 0 and the highest-risk category set to 1. This transformation assumes equal spacing between response categories and was used solely for visualization purposes; it does not imply that categories represent equal increments of biosecurity risk. A heatmap was generated using the pheatmap package (version 1.0.12) to visualize the distribution of biosecurity risk scores across farms for each survey question. Four continuous variables were excluded from the heatmap (years of experience of the main farm manager, number of persons employed, age of oldest poultry house, and age of newest poultry house), as they are not amenable to the 0 to 1 categorical risk scoring, resulting in 36 questions included in the heatmap.

To identify biosecurity variables associated with differences between intensive and extensive farms, Bayesian logistic regression with a regularized horseshoe prior was employed using the rstanarm package (version 2.32.2) in R. Production system was specified as the binary outcome (intensive = 0, extensive = 1), and the 28 selected biosecurity variables were entered as independent variables. The horseshoe prior is suitable for sparse high-dimensional settings, shrinking weakly supported coefficients toward zero while allowing strongly supported coefficients to remain large (Piironen and Vehtari, 2017). The prior was specified with a global scale of 0.075, half-Cauchy local and global scale parameters (1 degree of freedom), and Student-t regularization of the largest coefficients with 4 degrees of freedom and a scale of 2.5. Full settings are given in Supplementary Material C. Four chains were run for 4,000 iterations each, with the first 2,000 used as warmup, resulting in 8,000 posterior samples for inference. No thinning was applied. Categorical independent variables were modeled as unordered factors with dummy coding, with category 1 serving as the reference level; years of relevant experience of the main farm manager and number of persons employed on the farm were treated as continuous independent variables. Sampling was performed with adapt_delta set to 0.9999 to ensure convergence, given the combination of a relatively small extensive farm group (*n* = 15), a high-dimensional parameter space, and strong shrinkage priors. Convergence diagnostics were consistent with convergence: all R̂ values rounded to 1.00, and effective sample sizes were well above the threshold of 400 (minimum 2,825 among the model coefficients and 2,280 for the log-posterior). Model fit was assessed using posterior predictive checks and Pareto-smoothed importance-sampling leave-one-out **(PSIS-LOO)** cross-validation via the loo package (version 2.9.0) in R, with Pareto k diagnostics used to identify influential observations (Vehtari et al., 2017; see Supplementary Material C for full model diagnostics). As an additional model check, an approximate PSIS-LOO cross-validated area under the receiver operating characteristic curve **(AUC)** was calculated from leave-one-out predicted probabilities (Supplementary Material C). Associations were summarized using posterior means, 95% credible intervals (**CrI**), and posterior probabilities of direction, defined as the larger of P(β > 0) and P(β < 0). Exclusion of zero from the 95% CrI was not used as a binary classification rule; interpretation considered the magnitude, direction, and uncertainty of the posterior estimates jointly. For descriptive presentation, Fig. 3 shows the response distributions for the 3 categorical variables with the largest absolute posterior mean coefficients. Full coefficient summaries, including the continuous farm characteristics, are given in Supplementary Material D.

To assess whether the temporal spread of the intensive farm data (2015 to 2023) influenced the results, 3 complementary sensitivity analyses were conducted using a 2022 cut point. This cut point was chosen because all 15 extensive farms were surveyed after 2022 (December 2023 to May 2024); a 2022 boundary therefore places every extensive farm in the recent period while retaining 16 intensive farms in that period for comparison. These were a comparison of response-category distributions between intensive surveys collected before versus from 2022 onward, the full model fitted to all 78 farms with survey period (pre-2022 versus 2022 onward) added as a covariate, and a recent-period model restricted to the temporally overlapping subsample of the 16 intensive farms surveyed from 2022 onward together with all 15 extensive farms (*n* = 31). Details are given in Supplementary Material E. Additionally, prior and model sensitivity analyses were conducted to assess robustness to the horseshoe global scale parameter and the exclusion of structurally system-linked variables (see Supplementary Material F).

### Ethical considerations

This study was conducted within the BIOSECURE project, which received ethical approval from the Ethical Committee of the Faculty of Political and Social Sciences at Ghent University (approval number 2023-44). It involved collection of farm management data through questionnaires. Informed consent was obtained from all participating farmers, either directly prior to farm visits (extensive farms) or through the Biocheck.UGent platform (intensive farms). In addition, consent procedures for the extensive farm visits were verified with Utrecht University’s data protection officer prior to data collection. Farm identities were anonymized prior to analyses and reporting.

## Results

Biosecurity was compared across 78 farms (63 intensive, 15 extensive).

Intensive and extensive farms did not separate into distinct groups, and no consistent system-level patterns emerged. Instead, scores varied widely among farms within both systems: all 36 categorical variables in the heatmap showed more than 1 response category among intensive farms, and 34 of 36 did so among extensive farms (Fig. 1). Among extensive farms, the only 2 variables without variation were stocking density, where all farms were at or below 33 kg/m², and the number of steps in which the house was emptied during depopulation. Several variables showed consistently high risk scores in both intensive and extensive systems (visible as dark red rows across both groups in Fig. 1), indicating shared areas of elevated risk regardless of production system. These included cleaning and disinfection of feed silos, feed delivery frequency, poultry-free period requirements for visitors, and proximity to standing or flowing water. Of these, water proximity and feed delivery frequency reflect fixed environmental or structural characteristics, whereas the other measures are related to farm management practices.

**Fig. 1:**
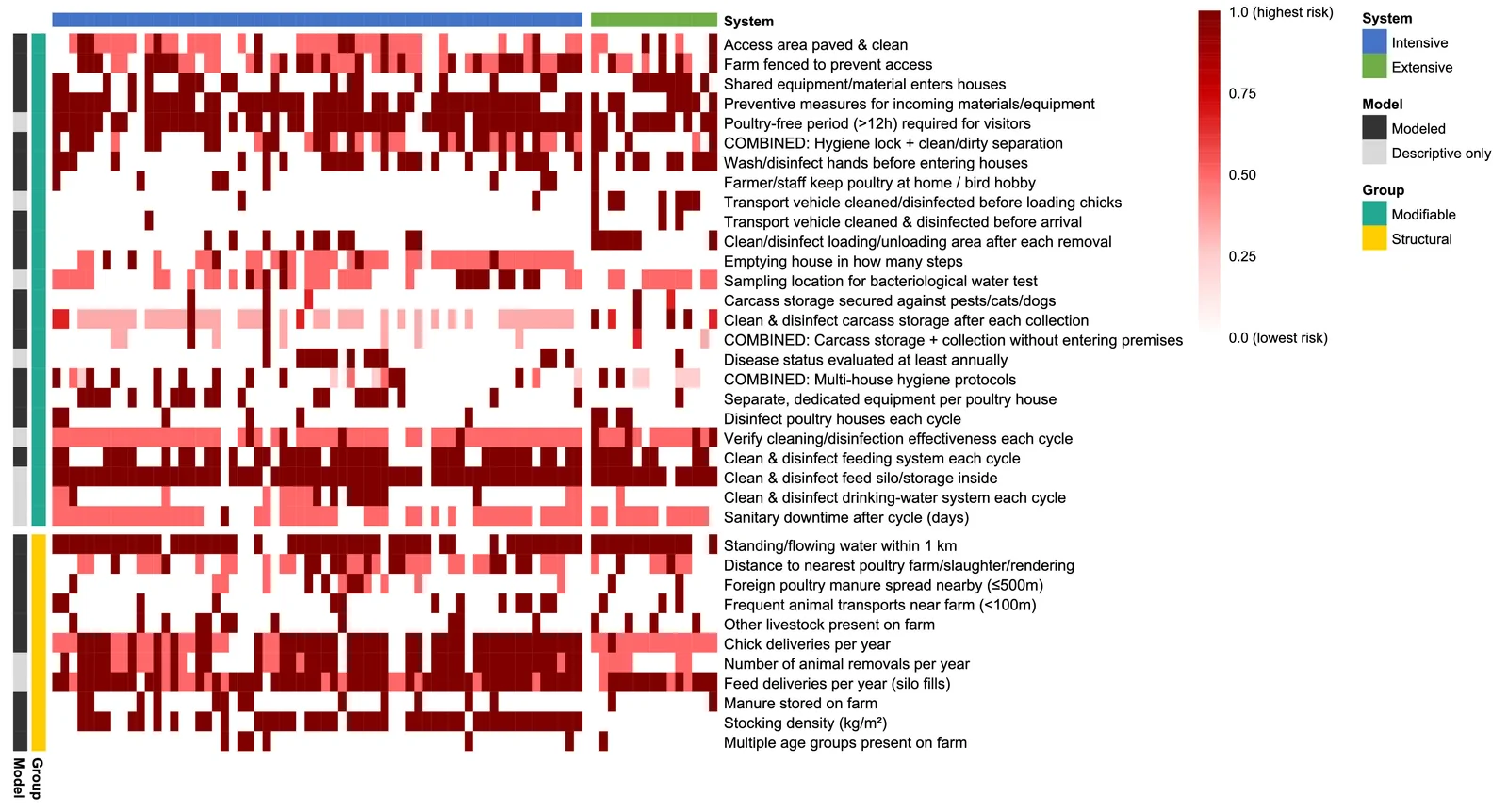
Heatmap of normalized (0 to 1 scaled) biosecurity risk scores across 78 farms (x-axis) for each of the 36 categorical survey variables (y-axis). Colors range from white (lowest risk, 0.0) to dark red (highest risk, 1.0). Farms are grouped by production system: intensive (*n* = 63, blue) and extensive (*n* = 15, green). Scores represent relative biosecurity risk based on ordinal response categories and are scaled for visualization purposes only. Variables labeled “COMBINED” represent merged survey questions assessing related practices. The side annotation bars show, for each variable, whether it is modifiable through farm management (Modifiable, teal) or reflects structural or environmental characteristics not under direct farm control (Structural, yellow), based on the authors' classification, and whether it was included in the Bayesian regression model (Modeled, dark gray) or retained for descriptive visualization only (Descriptive only, light gray). These 36 categorical variables are all harmonized variables except the 4 continuous ones, which were excluded because the heatmap represents categorical risk scores. Of the 36 variables shown, 26 were included in the model and 10 were used for descriptive visualization only; the model additionally included 2 continuous variables, years of experience of the main farm manager and number of persons employed on the farm, that are not displayed in this heatmap (28 model variables in total). The other 2 continuous variables, the ages of the oldest and newest poultry house, were not modeled and are reported in Supplementary Material B.

Among the 28 variables, the 3 categorical variables with the largest absolute posterior mean coefficients were: carcass storage cleaning, stocking density, and chick delivery frequency (Fig. 2). Because production system was modeled with intensive farms coded as 0 and extensive farms as 1, coefficients describe the association between each response category and production system after accounting for the other variables in the model, rather than a causal effect of production system on biosecurity. Relative to the reference category, negative coefficients indicate an association with intensive farms and positive coefficients indicate an association with extensive farms.

**Fig. 2:**
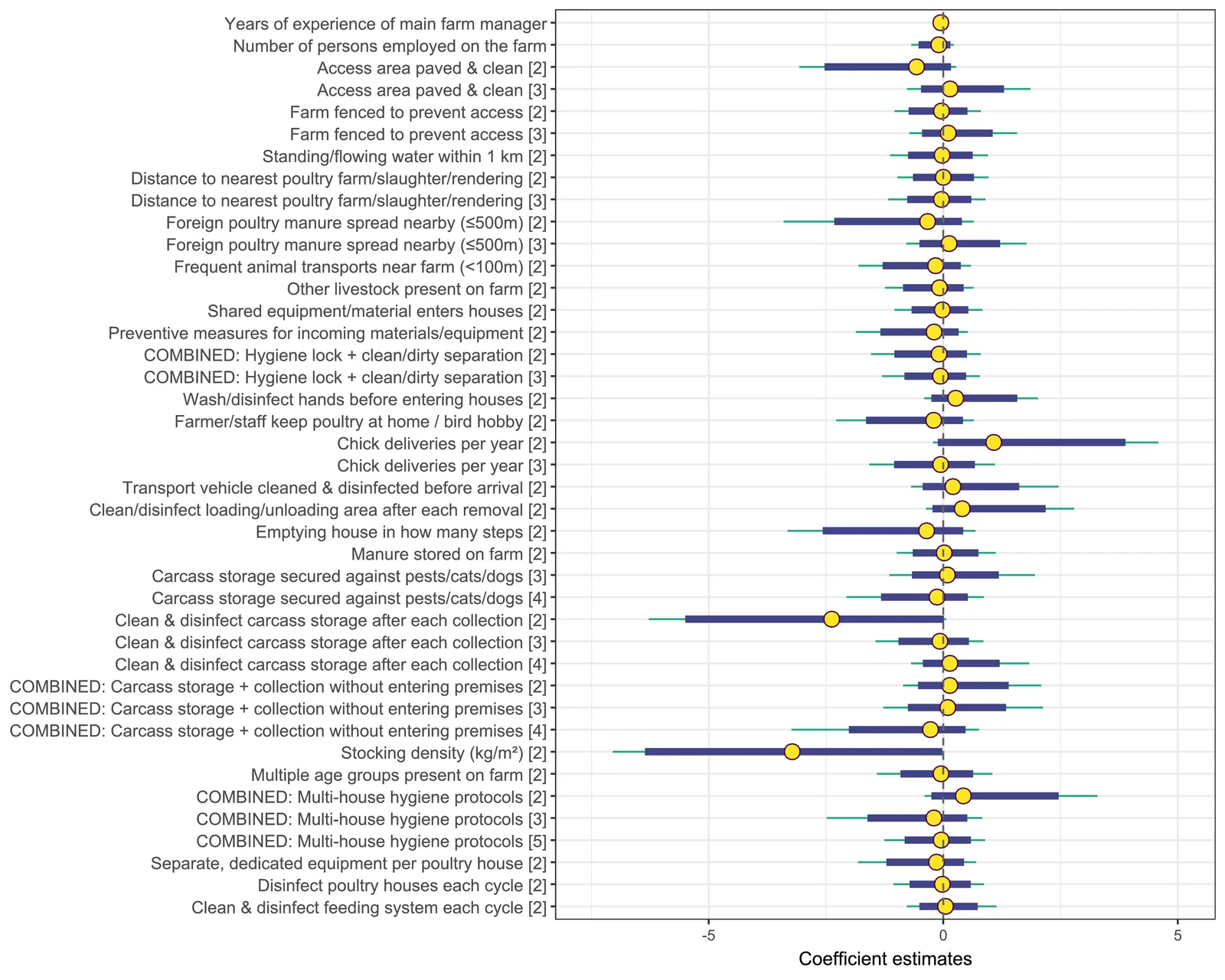
Coefficient estimates from the Bayesian logistic regression model with horseshoe priors for the 28 highly pathogenic avian influenza-related variables, based on 78 broiler farms (intensive, *n* = 63; extensive, *n* = 15). Yellow dots represent posterior means (see Supplementary Material D for full coefficient output). For each coefficient, the thick dark blue bar denotes the 90% credible interval and the thinner green line extending beyond it denotes the 95% credible interval. Values in brackets indicate the coded response category, where higher values correspond to higher biosecurity risk.

The remaining 23 categorical variables and the number of persons employed had posterior means near zero with wide credible intervals and should therefore not be interpreted as demonstrating similarity between systems. Manager experience had a small per-year coefficient (posterior mean –0.05 per year, 95% CrI: –0.14 to 0.01; posterior probability of a negative coefficient 0.94; Supplementary Material D).

The approximate PSIS-LOO cross-validated AUC was 0.94; given the small sample and the variables structurally related to production system, this reflects separation of the farms in this dataset rather than generalizable predictive performance.

To further illustrate the 3 categorical variables with the largest absolute posterior mean coefficients, their distributions by production system are shown in Fig. 3 (panels A–C). For carcass storage cleaning and disinfection after each collection, respondents selected 1 of 4 options: “Always,” “Sometimes,” “Never,” or “No Separate Carcass Storage,” with “Always” as the reference category. The posterior mean coefficient for “Sometimes” cleaning was –2.38 (95% CrI: –6.28 to 0.06; posterior probability of a negative coefficient 0.93), indicating that this response category was more common among intensive than extensive farms, although the 95% CrI included zero. The coefficients for “Never” and “No Separate Carcass Storage” were close to zero with wide credible intervals, providing limited evidence about these categories. Descriptive results (Fig. 3A) showed that 61.9% (39/63) of intensive farms reported cleaning “Sometimes”, whereas none of the extensive farms did (0/15); instead 60.0% (9/15) of extensive farms reported “Always” cleaning.

**Fig. 3:**
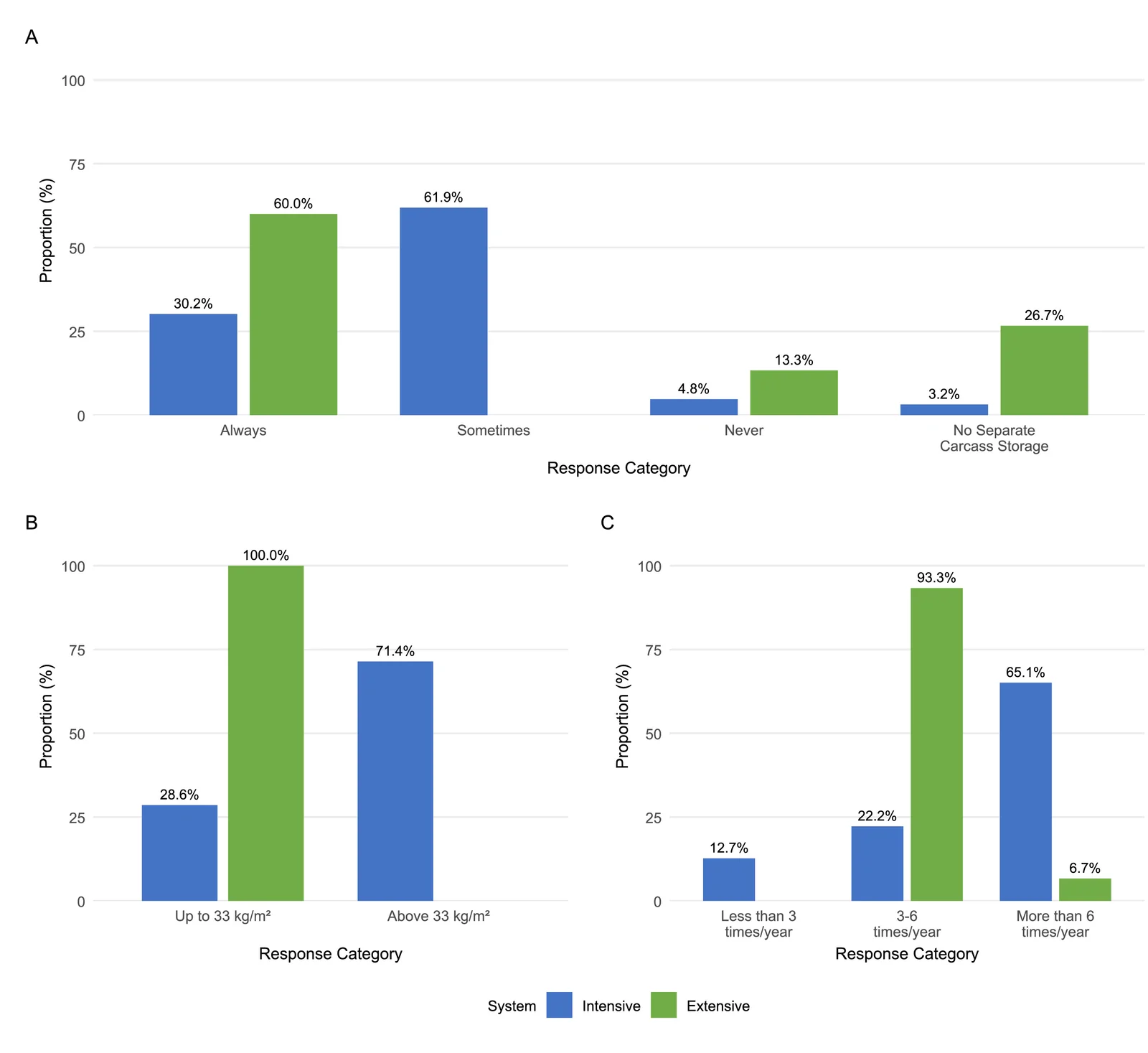
Distribution of the 3 categorical variables with the largest absolute posterior mean coefficients in the regression analysis (Fig. 2): (A) carcass storage cleaning and disinfection practices, (B) stocking density, and (C) chick delivery frequency, stratified by production system (intensive, *n* = 63; extensive, *n* = 15). Bars represent the proportion of farms within each response category; the corresponding numbers of farms are given in Supplementary Material B. For panel A, response categories: “Always” = cleaning after each carcass collection; “Sometimes” = intermediate frequency; “Never” = no cleaning; “No Separate Carcass Storage” = no separate carcass storage reported. For panel B, stocking density represents the final weight-based density of animals in the poultry house (kg/m²). For panel C, categories represent the number of times per year chicks are delivered to the farm.

Stocking density represents the final weight-based density of animals in the poultry house, and is a defining structural characteristic of the production system rather than a management characteristic. With “up to 33 kg/m²” as the reference category, the model estimated a posterior mean coefficient of –3.22 (95% CrI: –7.05 to 0.02; posterior probability of a negative coefficient 0.96) for densities above 33 kg/m², although the 95% CrI included zero. This negative coefficient indicates that higher stocking densities were less common on extensive farms. As shown in Fig. 3B, all extensive farms (15/15) maintained densities up to 33 kg/m², whereas 71.4% (45/63) of intensive farms exceeded this threshold.

Chick delivery frequency represents the number of chick placements per farm per year. “Less than 3 times” served as the reference category, with “3 to 6 times” and “More than 6 times” representing moderate and high delivery frequencies, respectively. The model estimated a posterior mean coefficient of +1.08 (95% CrI: –0.22 to 4.59; posterior probability of a positive coefficient 0.80) for the “3 to 6 times” category, indicating that extensive farms were more often within this moderate delivery range, although directional support was weaker than for the other 2 variables and the 95% CrI included zero. The coefficient for “More than 6 times” was near zero, indicating no clear system-level difference for this category. Descriptive results (Fig. 3C) showed that 93.3% (14/15) of extensive farms operated at 3 to 6 deliveries per year, whereas 65.1% (41/63) of intensive farms reported more than 6 deliveries per year.

Temporal sensitivity analyses indicated broadly stable patterns overall among intensive farms, although several variables showed descriptive changes between periods, particularly stocking density, emptying the house in multiple steps, cleaning and disinfection of the loading area, and manager experience (Supplementary Material E). The proportion above 33 kg/m² declined from 78.7% (37/47) before 2022 to 50.0% (8/16) thereafter. Because all extensive farms were surveyed from 2022 onward, the model could not fully separate temporal and system differences for this variable. Accordingly, the posterior mean coefficient for densities above 33 kg/m² (relative to the up to 33 kg/m² reference) attenuated from –3.22 to –1.31 when survey period was included as a covariate, whereas the carcass storage cleaning association remained consistent (–2.38 and –2.82 before and after adjustment; Supplementary Material E). This attenuation is consistent with the structural correlation between survey period and production system rather than indicating the absence of a stocking-density difference, and is further supported by the large coefficient estimated for the survey period covariate (3.96, 95% CrI: 0.01 to 8.03). Prior and model sensitivity analyses produced broadly similar estimates for the 3 variables shown in Fig. 3 across the prior settings examined, and the carcass storage cleaning association remained consistent after exclusion of the structurally system-linked variables (Supplementary Material F).

## Discussion

This study aimed to identify variation in biosecurity implementation among Dutch broiler farms and to evaluate whether such variation is systematically associated with production system. By comparing 28 HPAI-related biosecurity measures between 63 intensive and 15 extensive farms using a harmonized subset of Biocheck.UGent, we found limited evidence for systematic differences between production systems, with substantial variation in risk scores within both groups. The largest estimated differences between systems involved carcass storage cleaning and disinfection, stocking density, and chick delivery frequency, although the statistical support for these differences varied. Near-zero coefficients could in principle also arise if biosecurity varied little between farms overall, leaving little for the model to detect. However, the substantial between-farm variation argues against this explanation; instead, the observed variation appears to be distributed across individual farms rather than aligned with production system. This interpretation, based on the response distributions and regression estimates rather than formally estimated variance components, should be viewed cautiously. With only 15 extensive farms and a relatively large number of variables, coefficient estimates were imprecise despite reliable sampling, as reflected by the wide credible intervals, and modest system-level differences cannot be excluded. These findings highlight the importance of considering farm-specific weaknesses when developing biosecurity recommendations, rather than assuming that risks are aligned primarily with production system.

Several measures showed consistently high risk scores across both intensive and extensive systems (Fig. 1), including cleaning and disinfection of feed silos, feed delivery frequency, poultry-free period requirements for visitors, and proximity to standing or flowing water. Of these, water proximity and feed delivery frequency reflect environmental or structural characteristics, whereas feed silo cleaning and the visitor poultry-free period represent shared areas of suboptimal implementation across farms. This pattern suggests that certain biosecurity weaknesses are common across the sector regardless of production system type, whereas many other practices vary substantially between farms (Vougat Ngom et al., 2025). The shared weaknesses identified across both production systems provide concrete priorities for sector-wide improvement. Possible explanations for poor implementation of feed silo cleaning include limited perceived benefit, concerns that wet cleaning may introduce moisture and promote mold growth (Souillard et al., 2024), and practical and occupational-safety constraints associated with thorough silo cleaning, including working at height or, depending on silo design and cleaning procedures, entry into a confined space. Likewise, a poultry-free period of more than 12 h may be difficult to verify and impractical for veterinarians and advisors visiting several farms per day. More generally, practical constraints, perceived effectiveness, time requirements, costs, and farm infrastructure can influence whether recommended biosecurity measures are consistently applied (Souillard et al., 2024; Castillo et al., 2026). Improving implementation is therefore unlikely to depend on awareness alone. Interventions could combine clear explanation of the epidemiological rationale for individual measures with feasible farm-specific procedures, repeated reinforcement by veterinarians and farm advisors, and sector-level support. These can include, for example, silo-cleaning services offered by feed suppliers or coordinated visit planning. Farm-tailored management cannot, however, remove structural exposures such as proximity to water or the need for feed deliveries.

Among the categorical variables highlighted in Fig. 3, carcass storage cleaning was the only directly modifiable management practice and showed the most consistent estimates across the sensitivity analyses (Supplementary Materials E and F). Among farms that reported a cleaning frequency, extensive farms more often reported cleaning and disinfection after each collection (9 of 11, compared with 19 of 61 intensive farms). This difference should nevertheless be interpreted cautiously because responses concerning the presence of separate carcass storage were inconsistent for several extensive farms (4 of 15 reported no separate storage, of which 3 reported a dedicated storage elsewhere in the survey), indicating that differences in how carcass storage was configured or interpreted may have influenced responses. Under the Dutch poultry chain quality scheme (IKB Kip), carcass storage may be integrated into the farm building rather than provided as a physically separate facility, which may have contributed to different interpretations of what constituted “separate” carcass storage (Avined, 2026). Previous research has identified lack of advice or training as one reason for not cleaning carcass storage after each collection (Souillard et al., 2024), suggesting that this may be an area where clearer guidance could support implementation. Inadequate carcass management may allow persistence of pathogens and attract scavengers or pests, and carcass collection also involves vehicles visiting multiple farms, providing potential indirect pathways for avian influenza virus introduction (Fathelrahman et al., 2020; Souillard et al., 2024). These pathways were not assessed directly in the present study. These findings suggest that differences between systems may reflect a combination of infrastructure and management practices (Guinat et al., 2020; Mallioris et al., 2023; Islam et al., 2024), reinforcing the value of tailored biosecurity plans and on-site coaching.

Stocking density was a clear structural difference between production systems: the majority of intensive farms exceeded 33 kg/m², compared to none of the extensive farms, consistent with EU-defined production characteristics (Council of the European Union, 2007; European Commission, 2008). The lower proportion above 33 kg/m² among intensive farms surveyed from 2022 onward may reflect the adoption of systems with slower-growing breeds and reduced stocking density in the Netherlands (Slegers et al., 2024). However, because the intensive farms constituted a convenience sample, this temporal pattern cannot be distinguished from differences in sample composition and should not be interpreted as evidence of a sector-wide change. Stocking density is not itself a route of virus introduction and should also be distinguished from farm or flock size. During the 2003 H7N7 epidemic in the Netherlands, larger farm size, measured by the number of birds and poultry houses, was associated with HPAI introduction, plausibly through a higher frequency of external contacts, whereas housing type was not (Thomas et al., 2005). Recent modeling of Dutch poultry farms showed that smaller farm sizes reduced simulated HPAI epidemic size (Chanchaidechachai et al., 2026). However, this effect did not fully offset the modeled increase in introduction risk and epidemic size associated with increasing numbers of outdoor farms. Once virus has entered a flock, higher bird density may affect within-flock transmission dynamics (Sims et al., 2016), although this was not evaluated in the present study. Thus, the marked difference in stocking density observed here should primarily be regarded as a structural characteristic of the production systems rather than evidence of poorer biosecurity or greater HPAI introduction risk in intensive farms.

Chick delivery frequency showed a weaker and more uncertain contrast between systems, consistent with differences in production cycle length. Intensive systems, with faster-growing breeds, require more frequent chick placements than extensive systems with longer production cycles (Singh et al., 2021). Although largely structurally determined, chick delivery involves vehicle and personnel movements that could provide opportunities for pathogen introduction. However, evidence specifically implicating chick delivery as an important route of HPAI introduction is limited; EFSA assessed the likelihood of HPAI introduction through intra-EU movements of live poultry, including day-old chicks, as unlikely (EFSA AHAW Panel, 2017). Moreover, frequent chick delivery should not in itself be interpreted as greater HPAI introduction risk, as risk will also depend on the biosecurity measures surrounding these contacts. For example, verification that chick transport vehicles had been cleaned and disinfected before loading was reported more often by intensive than extensive farms (61/63 compared with 8/15; Supplementary Material B), although this variable was not included in the model. Many of these practices are influenced by sector-level organization, such as hatchery logistics and transport routines, and are therefore partly outside the farmer’s direct control (Fornasiero et al., 2023).

The 3 contrasts therefore differ in both the strength of statistical support and their interpretation in relation to biosecurity. Stocking density was sensitive to adjustment for survey period, whereas chick delivery frequency attenuated after adjustment and showed the weakest directional support of the 3 (Supplementary Material E). Carcass storage cleaning provided the most consistent evidence of a potentially modifiable management difference, whereas stocking density and chick delivery frequency primarily reflected structural characteristics of the production systems. Their association with production system should therefore not in itself be interpreted as evidence of differences in HPAI introduction risk.

Taken together, these findings have practical implications. Production system alone appears to be a limited proxy for biosecurity implementation, and interventions may be more effective when they target farm-specific weaknesses (Vougat Ngom et al., 2025). For farmers, veterinarians, and other farm advisors, including advisors affiliated with feed companies, tools such as Biocheck.UGent can support this by benchmarking farms and identifying specific areas for improvement (Castillo et al., 2026), and veterinary coaching based on such assessments has improved biosecurity scores on Belgian poultry farms (Amalraj et al., 2024). For policymakers, this suggests complementing system-level regulations with support for farm-level implementation, including the shared weaknesses that can be addressed across production systems. Industry organizations have a role where measures require coordination beyond the individual farm, as factors such as chick delivery logistics, feed transport, and carcass collection are typically managed at sector level (Souillard et al., 2024).

At the same time, farm-specific biosecurity should be distinguished from exposure pathways that are inherent to, or strongly associated with, the production system. In particular, outdoor access may increase opportunities for direct or indirect exposure to wild birds and contaminated environments (Fusaro et al., 2024; Cullen et al., 2025). Such system-specific exposure was not captured by the harmonized set of measures used in the present comparison. Similar implementation of general biosecurity measures therefore does not imply equivalent HPAI exposure between production systems. Farm-level management can reduce these risks but cannot fully eliminate exposure associated with outdoor access, which requires complementary measures directed specifically at the relevant exposure pathways.

Previous research has often compared biosecurity across countries, regions, or poultry types (Van Limbergen et al., 2018), whereas direct comparisons between intensive and extensive broiler farms using harmonized, HPAI-relevant measures remain limited. Such studies reported differences between species, production types, and regions (Delpont et al., 2021) and lower implementation of some hygiene measures on free-range meat poultry farms (Souillard et al., 2024). The smaller system differences found here may reflect the shared national context, with both systems subject to the same chain quality requirements (Avined, 2026), and the covered runs of the extensive farms. In addition, the harmonized comparison excluded system-specific measures such as outdoor range management, which may have reduced the observed differences between systems. These studies also indicate that biosecurity implementation is associated with factors other than production type, consistent with the substantial within-system variation observed here. Our findings and the harmonized assessment approach used here are likely relevant to other countries where higher-welfare broiler systems with outdoor access are expanding, given attention to local system definitions and context.

Several strengths and limitations should be considered. Key strengths of this study include the use of a standardized, harmonized subset of Biocheck.UGent questions focused on HPAI-relevant biosecurity practices, which enabled consistent comparison within a single regulatory context. Data collection during farm visits reduced variation in interpretation compared with self-reported surveys. The combination of descriptive analysis and Bayesian regression allowed patterns and their uncertainty to be examined, and the sensitivity analyses assessed how individual estimates changed across temporal and model specifications (Supplementary Materials E and F).

A number of limitations must also be acknowledged. First, the assessment combined directly observable items with self-reported ones. Structural and infrastructural features, such as fencing, paved working areas, hygiene locks, and the presence of dedicated carcass storage, are directly observable, whereas practices that cannot be verified during a single visit, such as the frequency of cleaning and disinfection, drinking-water testing, and adherence to visitor protocols, rely on farmer reporting and may therefore introduce measurement error. Survey-based assessments may overestimate implementation levels due to discrepancies between reported and actual practices (Castillo et al., 2026). Differences in question framing between the surveys may also introduce differential measurement error, as illustrated by differences between questions assessing the presence versus use of hygiene measures within the multi-house hygiene composite (Supplementary Material A). For this variable, the analysis assumed that a measure that was available was also used, which may not hold on every farm. Because the intensive survey recorded availability, actual use on those farms can only have been equal to or lower than recorded, so some intensive farms may have been classified more favorably than their actual practice. Because this composite comprises multiple response categories, the direction and magnitude of the resulting bias cannot be determined. Second, farms were not randomly sampled, and participation may be associated with higher motivation or management quality, which could affect the observed differences between systems in either direction. Because the number of farmers approached for the extensive group was not recorded, the participation rate is unknown, and no data on non-participating farms, such as farm size or region, were available to assess whether such selection occurred. The representativeness of the intensive farm sample could not be fully assessed, as the Biocheck.UGent intensive broiler survey does not collect flock size. Province information was available for only 16 of 63 surveys (25.4%), all from Noord-Brabant, Gelderland, Friesland, and Utrecht. Other provinces, including Overijssel and Drenthe, which together account for over 22% of Dutch broiler farms (CBS, 2025), were not represented among farms with available province information. The limited geographic information prevented assessment of the regional representativeness of the intensive sample and warrants caution when interpreting location-dependent variables such as proximity to water and other poultry farms, as HPAI risk varies regionally with wild bird density and landscape (Schreuder et al., 2022). As a convenience sample from the Biocheck.UGent database, the intensive farms may also represent farmers with greater engagement in biosecurity assessment, which could bias the sample toward higher biosecurity awareness. Biosecurity levels among the intensive farms may therefore be higher than in the wider Dutch broiler sector, and the observed frequencies of specific weaknesses should not be treated as representative of that sector. Also, some intensive surveys may have been completed independently by farmers, potentially leading to more socially desirable responses and higher apparent compliance than direct observation would record, whereas all extensive farms were assessed on-site. Because the method of data collection for individual intensive submissions could not be established retrospectively, the direction and magnitude of any resulting reporting bias cannot be determined. Third, the relatively small number of extensive farms (*n* = 15) relative to the number of variables limits the information available for each coefficient, so estimates near zero should be read as inconclusive rather than as evidence of similarity.

Additional limitations include temporal differences between datasets; sensitivity analyses indicated largely stable patterns, although the stocking density association should be interpreted cautiously due to limited separation of temporal and system differences, and residual temporal confounding cannot be excluded (Supplementary Material E). The cross-sectional design captures practices at a single time point and does not reflect seasonal variation. Residual confounding cannot be excluded, because farms may differ in size, region, infrastructure, and management context. Furthermore, exclusion of system-specific questions during harmonization, such as those on outdoor range management, may have reduced the ability to capture certain differences in exposure between systems. Finally, risk scores were not linked to HPAI incidence, so the relationship between measured practices and actual disease risk could not be evaluated. Nevertheless, the observed variation in implementation and shared weaknesses among the surveyed farms can help identify priorities for biosecurity improvement. Future epidemiological studies linking specific biosecurity practices and system-specific exposure pathways to HPAI occurrence could help determine which measures are most relevant to introduction risk and whether their importance differs between production systems. Such studies will require sufficiently large datasets and careful consideration of potential confounding by farm, environmental, and production-system characteristics.

## Conclusions

In conclusion, most HPAI-related biosecurity measures showed overlapping response distributions between intensive and extensive broiler farms, with substantial variation among farms within each system. Of the largest contrasts, carcass storage cleaning was the only directly modifiable practice and the most consistent across the sensitivity analyses, whereas stocking density and chick delivery frequency mainly reflected structural characteristics of the systems. These findings indicate that production system alone does not adequately capture biosecurity performance in practice. Biosecurity improvement should therefore address both farm-specific weaknesses and shared implementation gaps across the sector. At the same time, similar implementation of general biosecurity measures does not remove system-specific exposure pathways, particularly those associated with outdoor access. Future research should validate these findings in larger and longitudinal studies, incorporate system-specific exposure pathways such as wild bird contact in outdoor systems, evaluate whether farm-tailored biosecurity interventions and on-site coaching lead to measurable improvements in implementation and disease prevention, and determine whether the shared weaknesses identified here are also present in other countries and production systems.

## Funding

This study was funded by the European Union’s Horizon Europe research and innovation program under Grant Agreement No. 101083923 (BIOSECURE Enhanced and cost-effective biosecurity in livestock production). Views and opinions expressed are those of the author(s) only and do not necessarily reflect those of the European Union or the European Research Executive Agency **(REA)**. Neither the European Union nor REA can be held responsible for them. The funders had no role in the design of the study; in the collection, analyses, or interpretation of data; in the writing of the manuscript, or in the decision to publish the results.

## Declaration of AI and AI-assisted technologies in the writing process

During the preparation of this work, the authors used ChatGPT (OpenAI, GPT-5.2) in order to improve the readability and language of the manuscript. After using this tool, the authors reviewed and edited the content as needed and take full responsibility for the content of the publication.

## Author contributions

Yasmin Bakhshi conceived and designed the study, collected the farm data and curated the dataset, contributed to the design of the statistical analysis and carried it out, interpreted the results, prepared the figures, and wrote the first draft. Egil A.J. Fischer contributed to the design of the statistical analysis, obtained the funding, and administered the project. Jan van den Broek contributed to the design of the statistical analysis and carried it out. J. Arjan Stegeman conceived and designed the study, supervised the work, obtained the funding, and administered the project. Francisca C. Velkers conceived and designed the study, collected the farm data and curated the dataset, contributed to the design of the statistical analysis, supervised the work, obtained the funding, and administered the project. All authors critically revised the manuscript for important intellectual content, approved the exact final version before publication, and agree to be personally accountable for their own contributions and for ensuring that any question about the accuracy or integrity of the work is investigated and resolved.

## Disclosures

The authors declare the following financial interests/personal relationships which may be considered as potential competing interests: Prof. J.A. Stegeman (on behalf of Utrecht University, Prof. J.A. Stegeman received a European Union’s Horizon Europe research and innovation program grant (Grant Agreement No. 101083923), which supported the PhD position of the first author) reports financial support was provided by Horizon Europe. The other authors declare that they have no known competing financial interests or personal relationships that could have appeared to influence the work reported in this paper.

## Data Availability

The analysis code and the aggregated data and analytical outputs are openly available in Zenodo at https://doi.org/10.5281/zenodo.20813673. The individual farm-level survey responses are not publicly available, as they could permit identification of participating farms.

## Appendix Supplementary materials

Supplementary Material A. Biocheck.UGent survey harmonization and variable selection.

Supplementary Material B. Descriptive statistics per variable by production system.

Supplementary Material C. Bayesian model diagnostics.

Supplementary Material D. Full coefficient output for the baseline model.

Supplementary Material E. Temporal sensitivity analysis.

Supplementary Material F. Prior and model sensitivity analyses.

Supplementary Material G. STROBE checklist for cross-sectional studies.

## Footnotes

Supplementary material associated with this article can be found, in the online version, at doi:10.1016/j.psj.2026.107951.
