# Supplementary material for: Biosecurity implementation in indoor and outdoor-access broiler production systems in the Netherlands
Source: Poult Sci. 2026 Oct 12;105(12):107951. doi: 10.1016/j.psj.2026.107951 (PMC13696177; doi:10.1016/j.psj.2026.107951)
Supplement: Supplementary file 1 [file mmc1.docx]

# Supplementary Material A. Biocheck.UGent survey harmonization and variable selection

*All 40 Biocheck.UGent survey variables after harmonization of the intensive (79 questions) and extensive (100 questions) broiler survey versions. Ten questions were excluded prior to this stage: nine due to low variability and one due to missing data (see Materials and Methods). Full English question wording reflects the harmonized version used in analysis. For composite variables, source questions are indicated in the variable code column. Response categories shown are the final coded values used in analysis; where original categories were merged due to low cell counts, the remark column of the working file was applied. Category 1 represents best biosecurity practice (lowest risk); higher numbers indicate progressively greater risk. Continuous variables (BK2, BK3, BK7, BK8) were excluded from the heatmap visualization but included in the Bayesian regression model where indicated. The baseline model (28 variables) comprised the subset selected based on HPAI-specific literature review and expert consensus (see Materials and Methods).* *During harmonization, the 2 survey versions were compared question by question to identify analogous items and reconcile differences (see Materials and Methods). Two component questions within the multi-house hygiene composite (COMP90COMP95COMP96COMP97) were found to differ in measurement framing. For COMP95 (extensive Q84 / intensive Q67), the intensive survey assessed whether a house hygiene lock was present, whereas the extensive survey assessed whether it was used. For COMP97 (extensive Q86 / intensive Q70), the intensive survey assessed whether handwashing was possible, whereas the extensive survey assessed whether handwashing or disinfection was performed. These differences concern 1 harmonized variable rather than 2 separate model variables. The composite was retained, but its interpretation requires caution, because the availability of infrastructure does not necessarily imply its actual use. These framing differences may introduce differential measurement error between production systems.*

| **No.** | **Biocheck.UGent category** | **Code** | **Full question (harmonized English)** | **Response categories (final coding; 1 = lowest risk)** | **Composite variable** | **Included in baseline model (28 variables)** |
| --- | --- | --- | --- | --- | --- | --- |
| 1 | Farm characteristics | **BK2** | How many years of relevant experience does the main farm manager have in broiler husbandry? (continuous variable) | Continuous (years) | No | **Yes** |
| 2 | Farm characteristics | **BK3** | How many persons are employed on the farm? (continuous variable) | Continuous (persons) | No | **Yes** |
| 3 | Farm characteristics | **BK7** | How old (in years) is the oldest building in which broilers are housed? (continuous variable) | Continuous (years) | No | No |
| 4 | Farm characteristics | **BK8** | How old (in years) is the newest building in which broilers are housed? (continuous variable) | Continuous (years) | No | No |
| 5 | A – Infrastructure & location | **INF9** | Is the exterior of the poultry house paved and clean, particularly in the working area (around access roads), e.g. removal of weeds, waste, and building materials? | 1 = Yes, it is completely paved and clean 2 = It is only partially paved and clean 3 = No | No | **Yes** |
| 6 | A – Infrastructure & location | **INF10** | Is the farm fully or partially fenced to prevent access by visitors and/or animals? | 1 = Yes, it is completely fenced 2 = It is only partially fenced 3 = No | No | **Yes** |
| 7 | A – Infrastructure & location | **INF11** | Is there standing or flowing water in the vicinity of the farm (i.e. within a radius of 1 km)? | 1 = No 2 = Yes | No | **Yes** |
| 8 | A – Infrastructure & location | **INF12** | What is the distance to the nearest poultry farm, slaughterhouse or rendering plant? | 1 = More than 1 km 2 = Between 500 m and 1 km 3 = Less than 500 m | No | **Yes** |
| 9 | A – Infrastructure & location | **INF13** | Is poultry manure from other farms spread on adjacent fields within 500 m of the farm? | 1 = Never 2 = Sometimes 3 = Often | No | **Yes** |
| 10 | A – Infrastructure & location | **INF14** | Do frequent (≥1 times per day) livestock transports occur along the road passing the farm (distance between road and farm <100 m, e.g. due to proximity of a slaughterhouse)? | 1 = No 2 = Yes | No | **Yes** |
| 11 | A – Infrastructure & location | **INF15** | Are other livestock species kept on the same farm premises? | 1 = No 2 = Yes | No | **Yes** |
| 12 | B – Farm organization | **ORG33** | Is equipment/materials shared with other farms that enters the broiler houses and/or comes into contact with the broilers (e.g. catching equipment)? | 1 = No 2 = Yes | No | **Yes** |
| 13 | B – Farm organization | **ORG34** | Are specific preventive measures taken upon delivery of materials/equipment to the farm (e.g. UV disinfection cabinet, disinfection with alcohol, soap and water)? | 1 = Yes 2 = No | No | **Yes** |
| 14 | C – Visitors & personnel | **BEZ36** | Is a poultry-free period of more than 12 hours required of all visitors before they can access the broiler houses? | 1 = Yes 2 = No | No | No |
| 15 | C – Visitors & personnel | **BEZ37BEZ39** | Is a farm hygiene lock present, and does it have a strict separation between the clean and dirty areas? [Composite: BEZ37 + BEZ39] | 1 = Yes, a hygiene lock is present with strict separation 2 = Yes, a hygiene lock is present, but without strict separation 3 = No, a hygiene lock is not present | Yes (BEZ37 + BEZ39) | **Yes** |
| 16 | C – Visitors & personnel | **BEZ41** | Do visitors/personnel wash or disinfect their hands before entering the broiler houses? | 1 = Yes 1 = NAT (shower facility present and always used: recoded to Yes for extensive farms) 2 = No | No | **Yes** |
| 17 | C – Visitors & personnel | **BEZ46** | Does the farmer or farm staff keep poultry at home, or do they have a bird-related hobby (e.g. bird hunting, pigeon breeding, cockfighting)? | 1 = No 2 = Yes | No | **Yes** |
| 18 | D – Chick purchase | **EEND52** | Is it verified whether the transport vehicles are cleaned and disinfected before the animals are loaded at the supplier? | 1 = Always 2 = Sometimes 2 = Never 2 = I don't know | No | No |
| 19 | D – Chick purchase | **EEND53** | How many times per year are chicks delivered to the farm? | 1 = Less than 3 times a year 2 = Between 3 and 6 times a year 3 = More than 6 times a year | No | **Yes** |
| 20 | E – Broiler removal | **AFV55** | Is the transport vehicle (including transport crates and containers) always cleaned and disinfected before arrival at the farm? | 1 = Always 2 = Sometimes 2 = Never 2 = I don't know | No | **Yes** |
| 21 | E – Broiler removal | **AFV58** | Are the loading and unloading areas cleaned and disinfected after each removal of animals? | 1 = Yes, cleaned and disinfected 2 = Only cleaned 2 = Not cleaned and disinfected | No | **Yes** |
| 22 | E – Broiler removal | **AFV59** | In how many steps is a broiler house completely emptied during depopulation? | 1 = In one step 2 = In two steps or more | No | **Yes** |
| 23 | E – Broiler removal | **AFV60** | How many times per year are the animals removed from the farm (depopulation frequency)? | 1 = Less than 6 times a year 2 = Between 6 and 12 times a year 3 = More than 12 times a year | No | No |
| 24 | F – Feed & water | **VOED64** | How many times per year are the feed silos filled or feed delivered to the farm? | 1 = Less than 5 times a year 1 = Between 5 and 20 times a year 2 = Between 21 and 35 times a year 3 = More than 35 times a year | No | No |
| 25 | F – Feed & water | **VOED69** | Where is the water sample taken for bacteriological analysis? | 1 = At both the source and the last drinker 2 = At the last drinker only 3 = At the source only 4 = From the tank in the house anteroom, or other location (e.g. first drinker) | No | No |
| 26 | G – Manure & carcasses | **MEST70** | Is manure stored on the farm premises? | 1 = No 2 = Yes | No | **Yes** |
| 27 | G – Manure & carcasses | **MEST75** ^a^ | Is the carcass storage sealed so that vermin, dogs, and cats cannot access it? | 1 = Yes, it is completely closed 2 = It is only partially closed 3 = No 4 = No separate carcass storage (not applicable) | No | **Yes** |
| 28 | G – Manure & carcasses | **MEST76** | Is the carcass storage cleaned and disinfected after each collection by the rendering company? | 1 = Always, after each collection 2 = Sometimes after collection 2 = No, only after every production round 3 = Never 4 = No separate carcass storage (not applicable) | No | **Yes** |
| 29 | G – Manure & carcasses | **MEST73MEST80** | Is there dedicated carcass storage on the farm, and can the rendering company collect carcasses without entering the farm premises? [Composite: MEST73 + MEST80] | 1 = Yes, dedicated storage AND rendering company can collect without entering farm (e.g. from public road or dirty road) 2 = Yes, dedicated storage BUT rendering company must enter farm 3 = No dedicated storage, but carcasses immediately processed AND rendering company can collect without entering farm 4 = No dedicated storage AND rendering company must enter farm | Yes (MEST73 + MEST80) | **Yes** |
| 30 | H – Disease management | **ZIEK83** | Is the disease status of the farm regularly evaluated (i.e. at least once per year, e.g. serology, slaughterhouse findings, trends)? | 1 = Yes 2 = No | No | No |
| 31 | H – Disease management | **ZIEK87** | What is the stocking density in the broiler house (based on end-weight of the animals)? | 1 = Up to 33 kg/m² 2 = Higher than 33 kg/m² | No | **Yes** |
| 32 | H – Disease management | **ZIEK88** | Are poultry groups of different ages present on the farm? | 1 = No, single-age flock 2 = Yes, different age categories (sorted by house or within one house) | No | **Yes** |
| 33 | I – Compartment measures | **COMP90COMP95COMP96COMP97** | Are hygiene and structural protocols followed on the farm premises where multiple broiler houses are present? [Composite: COMP90 + COMP95 + COMP96 + COMP97] | 1 = COMP90=1, or all three hygiene items answered yes where COMP90=2 (reference category).  2 = Hygiene-lock and clean/dirty separation items answered yes; handwashing item answered no.  3 = Hygiene-lock and handwashing items answered yes; clean/dirty separation item answered no.  4 = Hygiene-lock item answered yes; clean/dirty separation and handwashing items answered no.  5 = Hygiene-lock item answered no; the other hygiene items do not determine category assignment. | Yes (COMP90 + COMP95 + COMP96 + COMP97) | **Yes** |
| 34 | I – Compartment measures | **COMP100** | Is there clearly identifiable, separate material provided per broiler house? | 1 = Yes, separate material available for each poultry house 1 = Yes, but occasionally shared 2 = No | No | **Yes** |
| 35 | J – Cleaning & disinfection | **RND104** | Are the broiler houses disinfected after each production round? | 1 = Yes 2 = No | No | **Yes** |
| 36 | J – Cleaning & disinfection | **RND106** | Is the effectiveness of cleaning and disinfection verified after each production round (e.g. using a hygienogram, swabs, etc.)? | 1 = Always 2 = Sometimes 3 = Never | No | No |
| 37 | J – Cleaning & disinfection | **RND108** | Are the feeding systems (e.g. storage bins, feed augers, and chain feeders) cleaned and disinfected after each production round? | 1 = Yes, cleaned and disinfected after each production cycle 2 = Only cleaned after each production cycle 2 = Cleaned but not after each production cycle (occasionally) 2 = Never | No | **Yes** |
| 38 | J – Cleaning & disinfection | **RND109** | Is there a feed silo/storage and is it cleaned and disinfected from the inside after each production cycle? | 1 = Yes, cleaned and disinfected after each production cycle (or every 1–2 cycles) 2 = Only cleaned after each production cycle 2 = Cleaned and disinfected, but not after each production cycle (occasionally/yearly) 2 = Never | No | No |
| 39 | J – Cleaning & disinfection | **RND110** | Are the drinking water systems cleaned and disinfected (inside and outside) after each production round? | 1 = Always 2 = Sometimes 2 = Never | No | No |
| 40 | J – Cleaning & disinfection | **RND111** | How many days does the sanitary empty period last after each production cycle? | 1 = More than 8 days 2 = Between 3 and 8 days 2 = Less than 3 days | No | No |

^a^ MEST75: Note on model coding: Category 2 (partially closed) had no respondents in either production system; categories 1, 3, and 4 were retained as separate levels in the model (reference = category 1).

**Notes on Composite Variables**

Three harmonized variables were created by combining two or more original Biocheck.UGent survey questions that were interconnected (responses to one question determined the applicability of subsequent questions). The source questions and the resulting combined response categories are listed below for transparency.

**BEZ37BEZ39 : COMBINED: Hygiene lock + clean/dirty separation (farm level)**

**Combined question:** "Is a FARM hygiene lock present and does it have a strict separation between the clean and dirty areas?"

**BEZ37 (extensive Q27 / intensive Q64):** "Is er een bedrijfshygiënesluis aanwezig bij het betreden van het bedrijf?"

English: "Is a farm hygiene lock present (when entering the farm)?" | Yes=1, No=2

**BEZ39 (extensive Q29 / intensive Q65):** "Is er een strikte scheiding tussen het schone en het vuile gedeelte van de bedrijfshygiënesluis?"

English: "Is there a strict separation between the clean and dirty areas of the farm hygiene lock?" | Yes=1, No=2

**Combined response categories:**

**Category 1:** Yes, a hygiene lock is present with a strict separation (best practice)

**Category 2:** Yes, a hygiene lock is present, but without a strict separation

**Category 3:** No, a hygiene lock is not present (highest risk)

Rationale: The presence of a hygiene lock (BEZ37) and its physical clean/dirty separation (BEZ39) are interdependent, a lock without separation provides limited biosecurity benefit. The combined variable captures this hierarchy in a single ordinal score.

**MEST73MEST80 : COMBINED: Carcass storage + collection without entering premises**

**Combined question:** "Is there a dedicated carcass storage, and can the carcasses be collected by the rendering company without entering the farm premises?"

**MEST73 (extensive Q63 / intensive Q20):** "Is er een kadaveropslag?"

English: "Is there a carcass storage facility?" | Yes=1, Not relevant (carcasses immediately processed)=2, No=2

**MEST80 (extensive Q70 / intensive Q21):** "Kunnen de kadavers worden opgehaald door het destructiebedrijf, zonder dat ze daarbij het bedrijfsterrein betreden?"

English: "Can carcasses be collected by the rendering company without entering the farm premises (e.g., from the public road, or via the dirty road)?" | Yes=1, No=2

Note: The combined variable references both Q20 (MEST73) and Q21 (MEST80) of the intensive survey.

**Combined response categories:**

**Category 1:** Yes, dedicated storage AND carcasses can be collected without entering premises (best practice)

**Category 2:** Yes, dedicated storage, but carcasses CANNOT be collected without entering premises

**Category 3:** No dedicated storage, but rendering company does NOT need to enter the farm (clean road access)

**Category 4:** No dedicated storage AND carcasses cannot be collected without entering premises (highest risk)

Rationale: The availability of carcass storage (MEST73) and the ability to collect without breaching the farm perimeter (MEST80) together define the carcass biosecurity pathway. A farm with no storage and no perimeter-accessible collection represents the highest risk of vehicle-borne pathogen introduction.

Note on model coding: Categories 3 (n=1) and 4 (n=2) had low cell counts but were retained as separate levels in the model; the regularized horseshoe prior provides stability for sparse categories.

**COMP90COMP95COMP96COMP97 : COMBINED: Multi-house hygiene protocols**

**Combined question:** "Are the hygiene and structural protocols followed on the farm premises where multiple broiler houses are present?"

**COMP90 (extensive Q80a / intensive Q77):** "Zijn er meerdere pluimveestallen aanwezig op het bedrijf?"

English: "Are there multiple poultry houses on the farm?" | No=1, Single house with multiple interconnected compartments=2, Yes=2

**COMP95 (extensive Q84 / intensive Q67):** "Wordt er een hygiënesluis per stal gebruikt door bezoekers/personeel voordat zij de stallen betreden?"

English: "Is a hygiene lock used per house by visitors/staff before entering the houses?" | Yes, always used=1, No=2

Note: Intensive Q67 asks whether a compartment hygiene lock is present (infrastructure); extensive Q84 asks whether it is actually used (behavior). Similar intent, different framing.

**COMP96 (extensive Q85 / intensive Q68):** "Is er een strikte scheiding tussen het schone en het vuile gedeelte van de stalhygiënesluis?"

English: "Is there a strict separation between the clean and dirty areas of the house hygiene lock?" | Yes=1, No=2

**COMP97 (extensive Q86 / intensive Q70):** "Wassen of desinfecteren bezoekers en personeel hun handen voordat ze de pluimveestal binnengaan?"

English: "Do visitors and staff wash or disinfect their hands before entering the poultry house?" | Yes=1, No=2

Note: The intensive survey asks whether hand washing is possible (infrastructure present); the extensive survey asks whether it is actually done (behavior). For COMP95 and COMP97, a “yes” response refers to infrastructure availability in the intensive survey and reported use in the extensive survey. Availability does not necessarily imply use. Combining these items does not resolve this difference in measurement framing, which may introduce differential measurement error between production systems.

**Combined response categories (codes 1 to 5; 4 observed):**

**Category 1 (COMP90=1 OR [COMP90=2 AND COMP95=1 AND COMP96=1 AND COMP97=1]):** No multiple poultry houses present, or all three hygiene items answered yes. This is the reference category in the model.

**Category 2 (COMP90=2; COMP95=1; COMP96=1; COMP97=2):** Hygiene-lock and clean/dirty separation items answered yes; handwashing item answered no.

**Category 3 (COMP90=2; COMP95=1; COMP96=2; COMP97=1):** Hygiene-lock and handwashing items answered yes; clean/dirty separation item answered no.

**Category 4 (COMP90=2; COMP95=1; COMP96=2; COMP97=2):** Hygiene-lock item answered yes; clean/dirty separation and handwashing items answered no. This category had no respondents in either production system and is therefore not estimated in the model.

**Category 5 (COMP90=2; COMP95=2):** Hygiene-lock item answered no; the remaining hygiene items do not determine category assignment.

For COMP95 and COMP97, “yes” and “no” retain their survey-specific meanings concerning infrastructure availability or reported use, as explained above.

Note on model coding: Category 1 (no multiple poultry houses present, or all hygiene protocols followed) is the reference category. Category 4 (separation and hand washing not followed) had no respondents in either production system and therefore does not appear in the model. The Bayesian model used four factor levels: categories 1 (reference), 2 (except hand washing), 3 (separation not maintained), and 5 (no hygiene lock), as reported in Supplementary Material D.

Abbreviations: ext. = extensive broiler survey; int. = intensive broiler survey; Q = question number in the original survey instrument; n = number of farms in category.

# Variables Excluded Prior to Harmonization

*Ten Biocheck.UGent survey questions were excluded prior to the harmonization and variable selection stage due to low variability (9 questions) or missing data (1 question). A question was excluded if it met the low-variability threshold simultaneously in both production systems: for extensive farms (n = 15), at most one farm answered differently from the majority (≥93.3% agreement), and for intensive farms (n = 63), no more than four farms disagreed with the majority response (≥93.7% agreement). Table percentages use the total farm count per system. For MEST74, the low-variability criterion was assessed using valid non-NAT responses (see footnote b). NAT = Not Applicable To this farm; NAT responses are reported separately and excluded from the valid-response denominator. Category 1 = best biosecurity practice (lowest risk); higher numbers indicate progressively greater risk.*

| **No.** | **Biocheck.UGent category** | **Code** | **Full question (harmonized English)** | **Response categories (final coding; 1 = lowest risk)** | **Extensive (n = 15) n (%)** | **Intensive (n = 63) n (%)** |
| --- | --- | --- | --- | --- | --- | --- |
| 1 | Infrastructure, location and housing | **INF16** | Are any poultry species other than broilers kept on the same farm site? | 1 = No  2 = Yes | 15 (100.0%)  0 (0.0%) | 61 (96.8%)  2 (3.2%) |
| 2 | Infrastructure, location and housing | **INF21** | Is a rodent control program present on the farm (other than cats)? | 1 = Active program  2 = Reactive or none | 15 (100.0%)  0 (0.0%) | 59 (93.7%)  4 (6.3%) |
| 3 | Removal of broilers | **AFV54** | Are the transport vehicles (including crates and containers) empty on arrival at the farm? | 1 = Yes, always  3 = Never | 14 (93.3%)  1 (6.7%) | 62 (98.4%)  1 (1.6%) |
| 4 | Feed and water | **VOED62** | Does the feed truck driver have access to the poultry houses or area where direct contact with birds is possible? | 1 = Never  2 = Sometimes | 15 (100.0%)  0 (0.0%) | 61 (96.8%)  2 (3.2%) |
| 5 | Feed and water | **VOED63** | Are the feed silos or feed storage rooms completely sealed against water, birds, and vermin? | 1 = Yes  2 = No | 14 (93.3%)  1 (6.7%) | 62 (98.4%)  1 (1.6%) |
| 6 | Feed and water | **VOED68** | How often are bacteriological analyses of the drinking water performed? | 1 = At least once a year  2 = Every two years  3 = Less frequent than 2-yearly | 15 (100.0%)  0 (0.0%)  0 (0.0%) | 60 (95.2%)  1 (1.6%)  2 (3.2%) |
| 7 | Manure and carcass removal | **MEST72** | Is the manure removed via the dirty road/area?ᵃ | 1 = Yes  2 = No  NAT | 11 (73.3%)  4 (26.7%)  0 (0.0%) | 27 (42.9%)  3 (4.8%)  33 (52.4%) |
| 8 | Manure and carcass removal | **MEST74** | Is the carcass storage cooled/refrigerated?ᵇ | 1 = Yes  2 = No  NAT | 14 (93.3%)  0 (0.0%)  1 (6.7%) | 58 (92.1%)  3 (4.8%)  2 (3.2%) |
| 9 | Disease management | **ZIEK84** | How often are dead birds removed from the poultry houses? | 1 = Daily or more frequent  2 = Less than daily | 15 (100.0%)  0 (0.0%) | 61 (96.8%)  2 (3.2%) |
| 10 | Cleaning and disinfection | **RND101** | Are the poultry houses cleaned after every production cycle? | 1 = Yes  2 = No | 15 (100.0%)  0 (0.0%) | 61 (96.8%)  2 (3.2%) |

*ᵃ MEST72: In intensive farms, 33 responses (52.4%) were recorded as missing in the data provided by Biocheck.UGent. As this level of missing data precluded reliable between-system comparison, the variable was excluded on this basis rather than strictly on the low-variability criterion applied to the other nine variables.*

*ᵇ MEST74: Farms answering NAT had no dedicated carcass storage. When percentages are recalculated on valid (non-NAT) responses only, agreement is 100.0% (extensive) and 95.1% (intensive), both meeting the low-variability threshold.*

*Abbreviations: ext. = extensive broiler survey; int. = intensive broiler survey; n = number of farms in category; NAT = Not Applicable To this farm.*
