# Supplementary material for: Biosecurity implementation in indoor and outdoor-access broiler production systems in the Netherlands
Source: Poult Sci. 2026 Oct 12;105(12):107951. doi: 10.1016/j.psj.2026.107951 (PMC13696177; doi:10.1016/j.psj.2026.107951)
Supplement: Supplementary file 2 [file mmc2.docx]

**Supplementary Material B. Descriptive statistics per variable by production system**

*Frequency distributions (n and %) for all 40 harmonized variables, split by production system: intensive (n=63) and extensive (n=15). For continuous variables (BK2, BK3, BK7, BK8), mean (SD) and median [min–max] are shown. For categorical variables, n and % are the number and proportion of farms per response category. Where categories were merged for the regression model (due to low cell counts), a note is provided. Category 1 = lowest risk (best biosecurity practice); higher numbers = greater risk.*

| **No.** | **Code** | **Variable** | **Response category** | **INT n** | **INT %** | **EXT n** | **EXT %** | **Note** |
| --- | --- | --- | --- | --- | --- | --- | --- | --- |
|  |  |  |  | **Intensive (n=63)** | | **Extensive (n=15)** | |  |
| **Farm characteristics** | | | | | | | | |
|  |  |  |  | INT | EXT |  |  |  |
| **37** | **BK2** | Years of relevant experience of main farm manager (continuous) | **Mean (SD)** | 26.6 (SD 10.5) | 18.9 (SD 16.5) | *Continuous variable* |  |  |
|  |  |  | **Median [range]** | 25 [4–50] | 9 [1–54] |  |  |  |
| **38** | **BK3** | Number of persons employed on the farm (continuous) | **Mean (SD)** | 2.5 (SD 1.4) | 2.5 (SD 1.9) | *Continuous variable* |  |  |
|  |  |  | **Median [range]** | 2 [1–7] | 2 [1–7] |  |  |  |
| **39** | **BK7** | Age of oldest building housing broilers (years) (continuous) | **Mean (SD)** | 30.1 (SD 15.1) | 29 (SD 16.4) | *Continuous variable* |  |  |
|  |  |  | **Median [range]** | 25 [0–63] | 27 [1.5–54] |  |  |  |
| **40** | **BK8** | Age of newest building housing broilers (years) (continuous) | **Mean (SD)** | 10.3 (SD 9.5) | 13.8 (SD 13.3) | *Continuous variable* |  |  |
|  |  |  | **Median [range]** | 7 [0–48] | 8 [1.5–50] |  |  |  |
| **A – Infrastructure, location & housing** | | | | | | | | |
| **36** | **INF9** | Exterior of poultry house paved and clean (access roads) | 1 = Completely paved and clean | 24 | 38.1% | 9 | 60.0% |  |
|  |  |  | 2 = Only partially paved and clean | 31 | 49.2% | 3 | 20.0% |  |
|  |  |  | 3 = No | 8 | 12.7% | 3 | 20.0% |  |
| **35** | **INF10** | Farm fenced to prevent access by visitors/animals | 1 = Completely fenced | 16 | 25.4% | 6 | 40.0% |  |
|  |  |  | 2 = Only partially fenced | 23 | 36.5% | 5 | 33.3% |  |
|  |  |  | 3 = No | 24 | 38.1% | 4 | 26.7% |  |
| **34** | **INF11** | Standing or flowing water within 1 km of the farm | 1 = No | 13 | 20.6% | 2 | 13.3% |  |
|  |  |  | 2 = Yes | 50 | 79.4% | 13 | 86.7% |  |
| **33** | **INF12** | Distance to nearest poultry farm/slaughterhouse/rendering plant | 1 = More than 1 km | 26 | 41.3% | 8 | 53.3% |  |
|  |  |  | 2 = Between 500 m and 1 km | 24 | 38.1% | 5 | 33.3% |  |
|  |  |  | 3 = Less than 500 m | 13 | 20.6% | 2 | 13.3% |  |
| **32** | **INF13** | Poultry manure from other farms spread on adjacent fields within 500 m | 1 = Never | 50 | 79.4% | 13 | 86.7% | *0 extensive farms in category 2* |
|  |  |  | 2 = Sometimes | 8 | 12.7% | 0 | 0.0% |  |
|  |  |  | 3 = Often | 5 | 7.9% | 2 | 13.3% |  |
| **31** | **INF14** | Frequent livestock transport along road passing farm (<100 m) | 1 = No | 50 | 79.4% | 12 | 80.0% |  |
|  |  |  | 2 = Yes | 13 | 20.6% | 3 | 20.0% |  |
| **30** | **INF15** | Other livestock species kept on same farm premises | 1 = No | 51 | 81.0% | 11 | 73.3% |  |
|  |  |  | 2 = Yes | 12 | 19.0% | 4 | 26.7% |  |
| **B – Farm organization & goods supply** | | | | | | | | |
| **29** | **ORG33** | Equipment/materials shared with other farms | 1 = No | 44 | 69.8% | 7 | 46.7% |  |
|  |  |  | 2 = Yes | 19 | 30.2% | 8 | 53.3% |  |
| **28** | **ORG34** | Specific preventive measures taken upon delivery of materials | 1 = Yes | 14 | 22.2% | 7 | 46.7% |  |
|  |  |  | 2 = No | 49 | 77.8% | 8 | 53.3% |  |
| **C – Visitors & personnel** | | | | | | | | |
| **27** | **BEZ36** | Poultry-free period (>12 h) required of all visitors | 1 = Yes | 11 | 17.5% | 4 | 26.7% |  |
|  |  |  | 2 = No | 52 | 82.5% | 11 | 73.3% |  |
| **26** | **BEZ37BEZ39** | Farm hygiene lock present with strict clean/dirty separation (composite) | 1 = Hygiene lock present, strict separation | 27 | 42.9% | 10 | 66.7% |  |
|  |  |  | 2 = Hygiene lock present, no strict separation | 14 | 22.2% | 1 | 6.7% |  |
|  |  |  | 3 = No hygiene lock | 22 | 34.9% | 4 | 26.7% |  |
| **25** | **BEZ41** | Visitors/personnel wash or disinfect hands before entering | 1 = Yes (incl. NAT recoded to Yes) | 39 | 61.9% | 6 | 40.0% | *NAT responses recoded to 1 for extensive farms with shower facility (BEZ40)* |
|  |  |  | 2 = No | 24 | 38.1% | 9 | 60.0% |  |
| **24** | **BEZ46** | Farmer/staff keep poultry at home or have bird-related hobby | 1 = No | 55 | 87.3% | 14 | 93.3% |  |
|  |  |  | 2 = Yes | 8 | 12.7% | 1 | 6.7% |  |
| **D – Purchase of day-old chicks** | | | | | | | | |
| **23** | **EEND52** | Verification that transport vehicles are cleaned/disinfected before chicks loaded | 1 = Always / Yes | 61 | 96.8% | 8 | 53.3% |  |
|  |  |  | 2 = Sometimes / Never / Don't know / No | 2 | 3.2% | 7 | 46.7% |  |
| **22** | **EEND53** | Number of chick deliveries to the farm per year | 1 = Less than 3 times/year | 8 | 12.7% | 0 | 0.0% | *0 extensive farms in category 1* |
|  |  |  | 2 = Between 3 and 6 times/year | 14 | 22.2% | 14 | 93.3% |  |
|  |  |  | 3 = More than 6 times/year | 41 | 65.1% | 1 | 6.7% |  |
| **E – Removal of broilers** | | | | | | | | |
| **21** | **AFV55** | Transport vehicle always cleaned and disinfected before arrival | 1 = Always / Yes | 62 | 98.4% | 12 | 80.0% |  |
|  |  |  | 2 = Sometimes / Never / Don't know | 1 | 1.6% | 3 | 20.0% |  |
| **20** | **AFV58** | Loading/unloading areas cleaned and disinfected after each removal | 1 = Yes, cleaned and disinfected | 53 | 84.1% | 8 | 53.3% |  |
|  |  |  | 2 = Only cleaned / Not cleaned and disinfected | 10 | 15.9% | 7 | 46.7% |  |
| **19** | **AFV59** | Number of steps to completely empty a broiler house | 1 = In one step | 22 | 34.9% | 15 | 100.0% | *All extensive farms in category 1; categories 2 and 3 merged in regression model* |
|  |  |  | 2 = In two steps | 35 | 55.6% | 0 | 0.0% |  |
|  |  |  | 3 = In more than two steps | 6 | 9.5% | 0 | 0.0% |  |
| **18** | **AFV60** | Number of times per year animals are removed from the farm | 1 = Less than 6 times/year | 13 | 20.6% | 9 | 60.0% | *0 extensive farms in category 3* |
|  |  |  | 2 = Between 6 and 12 times/year | 10 | 15.9% | 6 | 40.0% |  |
|  |  |  | 3 = More than 12 times/year | 40 | 63.5% | 0 | 0.0% |  |
| **F – Feed & water** | | | | | | | | |
| **17** | **VOED64** | Number of times per year feed silos are filled or feed delivered | 1 = Less than 5 or between 5–20 times/year | 2 | 3.2% | 1 | 6.7% |  |
|  |  |  | 2 = Between 21 and 35 times/year | 13 | 20.6% | 3 | 20.0% |  |
|  |  |  | 3 = More than 35 times/year | 48 | 76.2% | 11 | 73.3% |  |
| **16** | **VOED69** | Location of water sample for bacteriological analysis | 1 = At both source and last drinker | 29 | 46.0% | 5 | 33.3% | *3 original categories retained; category 4 absent in both groups* |
|  |  |  | 2 = At the last drinker only | 23 | 36.5% | 10 | 66.7% |  |
|  |  |  | 3 = At the source only | 11 | 17.5% | 0 | 0.0% |  |
| **G – Removal of manure & carcasses** | | | | | | | | |
| **15** | **MEST70** | Manure stored on the farm premises | 1 = No | 50 | 79.4% | 13 | 86.7% |  |
|  |  |  | 2 = Yes | 13 | 20.6% | 2 | 13.3% |  |
| **12** | **MEST73MEST80** | Dedicated carcass storage AND rendering company can collect without entering farm (composite) | 1 = Dedicated storage + no farm entry needed | 54 | 85.7% | 13 | 86.7% | *Category 3 absent in intensive; category 4 absent in extensive* |
|  |  |  | 2 = Dedicated storage + farm entry required | 7 | 11.1% | 1 | 6.7% |  |
|  |  |  | 3 = No dedicated storage + no farm entry needed | 0 | 0.0% | 1 | 6.7% |  |
|  |  |  | 4 = No dedicated storage + farm entry required | 2 | 3.2% | 0 | 0.0% |  |
| **14** | **MEST75** | Carcass storage sealed against vermin, dogs and cats | 1 = Yes, completely closed | 60 | 95.2% | 13 | 86.7% | *Category 2 absent in both groups* |
|  |  |  | 3 = No | 1 | 1.6% | 1 | 6.7% |  |
|  |  |  | 4 = No separate carcass storage (N/A) | 2 | 3.2% | 1 | 6.7% |  |
| **13** | **MEST76** | Carcass storage cleaned and disinfected after each collection | 1 = Always, after each collection | 19 | 30.2% | 9 | 60.0% | *Category 2 absent in extensive* |
|  |  |  | 2 = Sometimes / Only after production round | 39 | 61.9% | 0 | 0.0% |  |
|  |  |  | 3 = Never | 3 | 4.8% | 2 | 13.3% |  |
|  |  |  | 4 = No separate carcass storage (N/A) | 2 | 3.2% | 4 | 26.7% |  |
| **H – Disease management** | | | | | | | | |
| **11** | **ZIEK83** | Disease status of farm regularly evaluated (≥1×/year) | 1 = Yes | 50 | 79.4% | 14 | 93.3% |  |
|  |  |  | 2 = No | 13 | 20.6% | 1 | 6.7% |  |
| **10** | **ZIEK87** | Stocking density in broiler house (end-weight basis) | 1 = Up to 33 kg/m² | 18 | 28.6% | 15 | 100.0% | *All extensive farms ≤33 kg/m²; original 11 categories collapsed to binary in regression model* |
|  |  |  | 2 = Higher than 33 kg/m² | 45 | 71.4% | 0 | 0.0% |  |
| **9** | **ZIEK88** | Poultry groups of different ages present on the farm | 1 = No, single-age flock | 57 | 90.5% | 14 | 93.3% |  |
|  |  |  | 2 = Yes (different age groups by house or within house) | 6 | 9.5% | 1 | 6.7% |  |
| **I – Measures between compartments** | | | | | | | | |
| **8** | **COMP90COMP95COMP96COMP97** | Hygiene and structural protocols followed where multiple broiler houses present (composite) | 1 = No multiple houses, OR all protocols followed | 44 | 69.8% | 8 | 53.3% | *Category 3 absent in extensive; category 4 absent in both groups* |
|  |  |  | 2 = Protocols followed except hand washing | 4 | 6.3% | 5 | 33.3% |  |
|  |  |  | 3 = Protocols followed except separation | 4 | 6.3% | 0 | 0.0% |  |
|  |  |  | 5 = No hygiene lock | 11 | 17.5% | 2 | 13.3% |  |
| **7** | **COMP100** | Clearly identifiable, separate material provided per broiler house | 1 = Yes (or occasionally shared) | 42 | 66.7% | 14 | 93.3% |  |
|  |  |  | 2 = No | 21 | 33.3% | 1 | 6.7% |  |
| **J – Cleaning & disinfection** | | | | | | | | |
| **6** | **RND104** | Broiler houses disinfected after each production round | 1 = Yes | 57 | 90.5% | 11 | 73.3% |  |
|  |  |  | 2 = No | 6 | 9.5% | 4 | 26.7% |  |
| **5** | **RND106** | Effectiveness of cleaning and disinfection verified after each round | 1 = Always | 7 | 11.1% | 1 | 6.7% |  |
|  |  |  | 2 = Sometimes | 52 | 82.5% | 10 | 66.7% |  |
|  |  |  | 3 = Never | 4 | 6.3% | 4 | 26.7% |  |
| **4** | **RND108** | Feeding systems cleaned and disinfected after each production round | 1 = Yes, cleaned and disinfected | 23 | 36.5% | 6 | 40.0% |  |
|  |  |  | 2 = Only cleaned / Not after each cycle / Never | 40 | 63.5% | 9 | 60.0% |  |
| **3** | **RND109** | Feed silo/storage cleaned and disinfected from inside after each cycle | 1 = Yes, cleaned and disinfected after each cycle | 3 | 4.8% | 1 | 6.7% |  |
|  |  |  | 2 = Only cleaned / Occasionally / Never | 60 | 95.2% | 14 | 93.3% |  |
| **2** | **RND110** | Drinking water systems cleaned and disinfected after each round | 1 = Always | 44 | 69.8% | 14 | 93.3% | *Categories 2 and 3 merged in the harmonized coding; variable not included in the regression model* |
|  |  |  | 2 = Sometimes | 10 | 15.9% | 1 | 6.7% |  |
|  |  |  | 3 = Never | 9 | 14.3% | 0 | 0.0% |  |
| **1** | **RND111** | Duration of sanitary empty period after each production cycle | 1 = More than 8 days | 16 | 25.4% | 3 | 20.0% | *Categories 2 and 3 merged in the harmonized coding; variable not included in the regression model* |
|  |  |  | 2 = Between 3 and 8 days | 46 | 73.0% | 12 | 80.0% |  |
|  |  |  | 3 = Less than 3 days | 1 | 1.6% | 0 | 0.0% |  |
