# Supplementary material for: Biosecurity implementation in indoor and outdoor-access broiler production systems in the Netherlands
Source: Poult Sci. 2026 Oct 12;105(12):107951. doi: 10.1016/j.psj.2026.107951 (PMC13696177; doi:10.1016/j.psj.2026.107951)
Supplement: Supplementary file 3 [file mmc3.docx]

# Supplementary Material C. Bayesian model diagnostics

The baseline model was fitted using Bayesian logistic regression with horseshoe priors (rstanarm, R). Convergence diagnostics were consistent with convergence: all R̂ values rounded to 1.00, and the minimum effective sample size (n_eff) was 2,825 among the model coefficients and 2,280 for the log-posterior, well above the threshold of 400. Model fit was assessed using LOO cross-validation (loo package) and posterior predictive checks (pp_check).

**C.1 Model Specification**

| **Setting** | **Value** |
| --- | --- |
| **Model type** | Bayesian logistic regression (binomial family, logit link) |
| **R package** | rstanarm (stan_glm) |
| **Outcome variable** | Production system type (0 = intensive, 1 = extensive) |
| **Predictors** | 28 HPAI-related biosecurity variables (see Supplementary Material A) |
| **Prior** | Horseshoe: global_scale = 0.075, slab_scale = 2.5, df = 1, global_df = 1, slab_df = 4 |
| **Chains** | 4 Markov chains |
| **Iterations** | 4,000 per chain (2,000 warmup) → 8,000 posterior samples |
| **adapt_delta** | 0.9999 |
| **Convergence criterion** | R̂ < 1.01; visual inspection of trace plots |
| **Model fit** | LOO cross-validation (loo package); posterior predictive checks (pp_check) |

**C.2 Convergence Summary**

All R̂ values rounded to 1.00 for all 44 monitored parameters (including mean_PPD and log-posterior), consistent with convergence across four chains. Monte Carlo standard errors (mcse) were 0.01 or less for most reported parameters; the largest values among the model coefficients were 0.03 for factor(EEND53)2, factor(MEST76)2 and factor(ZIEK87)2, while the log-posterior mcse was 0.19, all within acceptable limits. Effective sample sizes (n_eff) for the model coefficients ranged from 2,825 (factor(EEND53)2) to 8,691 (factor(INF10)2); the log-posterior effective sample size was 2,280. Full parameter-level diagnostics are in Table C.3 (landscape page). Analyses were run with a fixed seed (20240517), so these diagnostics are reproducible.

**C.3 LOO Cross-Validation**

Leave-one-out cross-validation was computed using Pareto-smoothed importance sampling (PSIS-LOO), accounting for the relative efficiency of the MCMC draws. Of the 78 observations, 77 (98.7%) had Pareto k values at or below 0.7. One observation had a Pareto k value marginally above this threshold (k = 0.704). The PSIS-LOO estimates were therefore retained and interpreted with appropriate caution.

**Pareto k diagnostics and refitting status**

| **Model** | **Problematic obs. (initial k > 0.7)** | **Action taken** | **Final diagnostic status** |
| --- | --- | --- | --- |
| **Baseline model** | 1 obs. (observation 2; k = 0.704) | None here; PSIS-LOO estimates retained. Exact refitting of this observation was carried out for the model comparison in Supplementary Material F and did not change that comparison. | 77 of 78 observations (98.7%) with k ≤ 0.7; maximum k = 0.704 |

**Final LOO metrics (PSIS-LOO with relative efficiency of the MCMC draws)**

| **Metric** | **Value** | **Interpretation** |
| --- | --- | --- |
| **elpd_loo** | −19.9 | Expected log pointwise predictive density |
| **SE of elpd_loo** | 3.2 | Standard error of elpd_loo |
| **p_loo** | 7.6 | Effective number of parameters |
| **looic** | 39.8 | LOO information criterion (−2 × elpd_loo); lower = better fit |
| **SE of looic** | 6.3 | Standard error of looic |
| **MCSE of elpd_loo** | NA | Not reported by the loo package when any Pareto k value exceeds 0.7, as is the case here (k = 0.704) |
| **Pareto k < 0.7** | 77 (98.7%) | Reliable PSIS-LOO estimates for 77 of 78 observations |
| **Pareto k ≥ 0.7** | 1 (1.3%) | One observation marginally above the threshold (k = 0.704) |

**C.4 Posterior Predictive Check**

The observed outcome distribution (dark line) closely overlaps the replicated distributions (green lines), indicating that the replicated marginal outcome proportions are compatible with the observed proportion.

Figure C1. Posterior predictive check: baseline model. Dark line = observed outcome distribution (y); green lines = replicated distributions (y_rep). Close overlap indicates that the replicated marginal outcome proportions are compatible with the observed proportion.

**C.5 PSIS Diagnostic Plot**

Seventy-seven of the 78 Pareto k values were at or below 0.7. One observation had a Pareto k value of 0.704, marginally exceeding the conventional diagnostic threshold. The k = 0.5 reference marks the boundary between good and moderate importance-sampling reliability.

Figure C2. PSIS diagnostic plot for the baseline model. Seventy-seven observations had Pareto k values at or below 0.7; one observation marginally exceeded this threshold (k = 0.704).

**C.6 Additional Model Check**

Because production system was the binary model outcome, an approximate Pareto-smoothed importance-sampling leave-one-out (PSIS-LOO) cross-validated area under the receiver operating characteristic curve (AUC) was calculated from leave-one-out predicted probabilities as an additional check on model fit. The AUC was 0.94, indicating good separation of intensive and extensive farms in this dataset. The leave-one-out probabilities were obtained by reweighting the posterior draws with Pareto-smoothed importance sampling, and the AUC was calculated as the probability that a randomly chosen extensive farm received a higher leave-one-out probability than a randomly chosen intensive farm. Given the small sample, the AUC should not be read as evidence of predictive performance in other farms or settings. This separation should be interpreted in the context of several variables that are structurally related to production system, particularly stocking density and chick delivery frequency, and does not imply that most individual biosecurity measures differed between systems.

Note: R̂ = potential scale reduction factor (Gelman & Rubin, 1992); n_eff = effective sample size; mcse = Monte Carlo standard error; elpd_loo = expected log pointwise predictive density; p_loo = effective number of parameters; looic = LOO information criterion (−2 × elpd_loo). n_eff values are reproducible under the fixed seed used here (20240517) but may differ under another seed or software version.

**Table C.3 Full MCMC Diagnostics: Baseline Model (28 variables)**

All reported R̂ values rounded to 1.00. n_eff from 8,000 posterior draws (4 chains × 2,000 post-warmup iterations).

| **Parameter** | **mcse** | **R̂** | **n_eff** | **Note** |
| --- | --- | --- | --- | --- |
| (Intercept) | 0.03 | 1.00 | 3,622 | n/a |
| Years of experience of main farm manager | 0.00 | 1.00 | 5,056 | Continuous |
| Number of persons employed on the farm | 0.00 | 1.00 | 6,166 | Continuous |
| Access area paved & clean [2] | 0.01 | 1.00 | 4,104 | n/a |
| Access area paved & clean [3] | 0.01 | 1.00 | 6,468 | n/a |
| Farm fenced to prevent access [2] | 0.00 | 1.00 | 8,691 | n/a |
| Farm fenced to prevent access [3] | 0.01 | 1.00 | 6,762 | n/a |
| Standing/flowing water within 1 km [2] | 0.01 | 1.00 | 8,392 | n/a |
| Distance to nearest poultry farm/slaughterhouse/rendering plant [2] | 0.00 | 1.00 | 8,095 | n/a |
| Distance to nearest poultry farm/slaughterhouse/rendering plant [3] | 0.01 | 1.00 | 7,917 | n/a |
| Foreign poultry manure spread nearby (≤500m) [2] | 0.02 | 1.00 | 4,608 | n/a |
| Foreign poultry manure spread nearby (≤500m) [3] | 0.01 | 1.00 | 7,148 | n/a |
| Frequent animal transports near farm (<100m) [2] | 0.01 | 1.00 | 5,658 | n/a |
| Other livestock present on farm [2] | 0.01 | 1.00 | 7,094 | n/a |
| Shared equipment/material enters houses [2] | 0.00 | 1.00 | 8,283 | n/a |
| Preventive measures for incoming materials/equipment [2] | 0.01 | 1.00 | 5,883 | n/a |
| COMBINED: Hygiene lock + clean/dirty separation [2] | 0.01 | 1.00 | 6,837 | n/a |
| COMBINED: Hygiene lock + clean/dirty separation [3] | 0.01 | 1.00 | 7,541 | n/a |
| Wash/disinfect hands before entering houses [2] | 0.01 | 1.00 | 5,313 | n/a |
| Farmer/staff keep poultry at home / bird hobby [2] | 0.01 | 1.00 | 5,408 | n/a |
| Chick deliveries per year [2] | 0.03 | 1.00 | 2,825 | n/a |
| Chick deliveries per year [3] | 0.01 | 1.00 | 6,694 | n/a |
| Transport vehicle cleaned & disinfected before arrival [2] | 0.01 | 1.00 | 5,603 | n/a |
| Clean/disinfect loading/unloading area after each removal [2] | 0.01 | 1.00 | 3,134 | n/a |
| Emptying house in how many steps [2] | 0.02 | 1.00 | 3,547 | n/a |
| Manure stored on farm [2] | 0.01 | 1.00 | 7,922 | n/a |
| Carcass storage secured against pests/cats/dogs [3] | 0.01 | 1.00 | 7,697 | n/a |
| Carcass storage secured against pests/cats/dogs [4] | 0.01 | 1.00 | 6,057 | n/a |
| Clean & disinfect carcass storage after each collection [2] | 0.03 | 1.00 | 2,857 | 95% CrI slightly includes 0 |
| Clean & disinfect carcass storage after each collection [3] | 0.01 | 1.00 | 7,776 | n/a |
| Clean & disinfect carcass storage after each collection [4] | 0.01 | 1.00 | 7,533 | n/a |
| COMBINED: Carcass storage + collection without entering premises [2] | 0.01 | 1.00 | 6,359 | n/a |
| COMBINED: Carcass storage + collection without entering premises [3] | 0.01 | 1.00 | 5,921 | n/a |
| COMBINED: Carcass storage + collection without entering premises [4] | 0.01 | 1.00 | 5,900 | n/a |
| Stocking density (kg/m²) [2] | 0.03 | 1.00 | 3,245 | 95% CrI slightly includes 0 |
| Multiple age groups present on farm [2] | 0.01 | 1.00 | 8,113 | n/a |
| COMBINED: Multi-house hygiene protocols [2] | 0.01 | 1.00 | 4,897 | n/a |
| COMBINED: Multi-house hygiene protocols [3] | 0.01 | 1.00 | 6,740 | n/a |
| COMBINED: Multi-house hygiene protocols [5] | 0.01 | 1.00 | 8,374 | n/a |
| Separate, dedicated equipment per poultry house [2] | 0.01 | 1.00 | 7,242 | n/a |
| Disinfect poultry houses each cycle [2] | 0.00 | 1.00 | 8,277 | n/a |
| Clean & disinfect feeding system each cycle [2] | 0.00 | 1.00 | 8,130 | n/a |
| mean_PPD | 0.00 | 1.00 | 8,321 | Fit diagnostic |
| log-posterior | 0.19 | 1.00 | 2,280 | n/a |

**Figure C3 Forest Plot: Baseline Model (28 HPAI-related variables)**

Posterior mean (dot), 90% credible interval (dark bar), and 95% credible interval (light bar) for all predictor coefficients. The strongest negative signals were observed for category 2 of cleaning and disinfecting carcass storage after each collection and category 2 of stocking density; however, both 95% CrIs slightly included zero. These variables are highlighted in the main text.

Figure C3. Forest plot: baseline model. All 28 HPAI-related variables. Dashed vertical line at zero. Dark bar = 90% CrI; light bar = 95% CrI; dot = posterior mean.
