# Supplementary material for: Biosecurity implementation in indoor and outdoor-access broiler production systems in the Netherlands
Source: Poult Sci. 2026 Oct 12;105(12):107951. doi: 10.1016/j.psj.2026.107951 (PMC13696177; doi:10.1016/j.psj.2026.107951)
Supplement: Supplementary file 4 [file mmc4.docx]

# Supplementary Material D. Full coefficient output for the baseline model

Posterior estimates (mean, median, SD, 95% credible intervals, and posterior probability of direction) from the Bayesian logistic regression model with a regularized horseshoe prior (rstanarm, R). Outcome: production system type (0 = intensive, 1 = extensive). The posterior probability of direction is the larger of P(β > 0) and P(β < 0); for the 2 continuous variables it refers to the per-unit coefficient. Positive coefficients indicate higher odds of extensive farming and negative coefficients lower odds, relative to the stated reference category and conditional on the other variables in the model; for the 2 continuous variables the coefficient refers to a 1-unit increase. All coefficients for the 28 variables are shown (41 coefficients due to factor levels), plus the intercept and mean_PPD. Posterior medians are reported for the 41 model coefficients; the mean is used as the point estimate throughout the manuscript, and the median is given here so that both summaries are available in one place.

The 3 categorical variables illustrated in Figure 3, ZIEK87 [2], MEST76 [2] and EEND53 [2], have the largest absolute posterior mean coefficients among the categorical variables. This descriptive ranking does not establish practical importance or certainty of association.

| **No.** | **Variable code** | **Variable description and response category (vs. reference category)** | **Biocheck.UGent** | **Mean** | **Median** | **SD** | **2.5% CrI** | **97.5% CrI** | **P(direction)** | **Note** |
| --- | --- | --- | --- | --- | --- | --- | --- | --- | --- | --- |
| **FARM CHARACTERISTICS** | | | | | | | | | | |
| 1 | BK2 | Years of relevant experience of the main farm manager in broiler husbandry [continuous] | n/a | -0.05 | -0.05 | 0.04 | -0.14 | 0.01 | 0.940 | Continuous predictor; no reference category |
| 2 | BK3 | Number of persons employed on the farm [continuous] | n/a | -0.10 | -0.03 | 0.22 | -0.68 | 0.22 | 0.662 | Continuous predictor; no reference category |
| **INFRASTRUCTURE, LOCATION AND HOUSING (INF)** | | | | | | | | | | |
| 3 | INF9 [2] | Access area paved & clean: partial (ref: fully paved & clean) | INF | -0.57 | -0.13 | 0.93 | -3.07 | 0.28 | 0.740 |  |
| 4 | INF9 [3] | Access area paved & clean: no (ref: fully paved & clean) | INF | 0.14 | 0.01 | 0.61 | -0.77 | 1.86 | 0.569 |  |
| 5 | INF10 [2] | Farm fenced to prevent access: partial (ref: fully fenced) | INF | -0.04 | -0.00 | 0.42 | -1.04 | 0.80 | 0.534 |  |
| 6 | INF10 [3] | Farm fenced to prevent access: no (ref: fully fenced) | INF | 0.11 | 0.01 | 0.53 | -0.72 | 1.57 | 0.556 |  |
| 7 | INF11 [2] | Standing/flowing water within 1 km: yes (ref: no) | INF | -0.03 | -0.00 | 0.48 | -1.13 | 0.95 | 0.513 |  |
| 8 | INF12 [2] | Distance to nearest poultry farm/slaughterhouse/rendering plant: 500m to 1 km (ref: >1 km) | INF | 0.00 | -0.00 | 0.44 | -0.98 | 0.97 | 0.503 |  |
| 9 | INF12 [3] | Distance to nearest poultry farm/slaughterhouse/rendering plant: less than 500m (ref: >1 km) | INF | -0.04 | -0.00 | 0.48 | -1.17 | 0.90 | 0.531 |  |
| 10 | INF13 [2] | Foreign poultry manure spread nearby (≤500m): sometimes (ref: never) | INF | -0.33 | -0.02 | 1.03 | -3.41 | 0.65 | 0.607 |  |
| 11 | INF13 [3] | Foreign poultry manure spread nearby (≤500m): often (ref: never) | INF | 0.12 | 0.01 | 0.59 | -0.79 | 1.77 | 0.560 |  |
| 12 | INF14 [2] | Frequent animal transports near farm (<100m): yes (ref: no) | INF | -0.16 | -0.02 | 0.56 | -1.81 | 0.59 | 0.589 |  |
| 13 | INF15 [2] | Other livestock present on farm: yes (ref: no) | INF | -0.08 | -0.01 | 0.44 | -1.24 | 0.65 | 0.545 |  |
| **FARM ORGANIZATION AND INCOMING GOODS (ORG)** | | | | | | | | | | |
| 14 | ORG33 [2] | Shared equipment/material enters houses: yes (ref: no) | ORG | -0.02 | -0.00 | 0.43 | -1.04 | 0.84 | 0.500 |  |
| 15 | ORG34 [2] | Preventive measures for incoming materials: no (ref: yes) | ORG | -0.20 | -0.03 | 0.58 | -1.86 | 0.52 | 0.614 |  |
| **VISITORS AND PERSONNEL (BEZ)** | | | | | | | | | | |
| 16 | BEZ37BEZ39 [2] | Hygiene lock + clean/dirty separation: partial (ref: full) | BEZ | -0.09 | -0.00 | 0.54 | -1.54 | 0.80 | 0.541 | COMBINED variable (BEZ37 + BEZ39) |
| 17 | BEZ37BEZ39 [3] | Hygiene lock + clean/dirty separation: no (ref: full) | BEZ | -0.06 | -0.01 | 0.46 | -1.31 | 0.78 | 0.539 | COMBINED variable (BEZ37 + BEZ39) |
| 18 | BEZ41 [2] | Wash/disinfect hands before entering houses: no (ref: yes) | BEZ | 0.26 | 0.04 | 0.61 | -0.40 | 2.02 | 0.654 |  |
| 19 | BEZ46 [2] | Farmer/staff keep poultry at home/bird hobby: yes (ref: no) | BEZ | -0.20 | -0.02 | 0.70 | -2.28 | 0.65 | 0.584 |  |
| **PURCHASE OF DAY-OLD CHICKS (EEND)** | | | | | | | | | | |
| 20 | EEND53 [2] | Chick deliveries per year: 3 to 6 times a year (ref: <3 times/year) | EEND | **1.08** | **0.43** | 1.39 | -0.22 | **4.59** | 0.802 | Shown in Figure 3; largest positive posterior mean coefficient among categorical variables |
| 21 | EEND53 [3] | Chick deliveries per year: more than 6 times a year (ref: <3 times/year) | EEND | -0.06 | -0.00 | 0.61 | -1.58 | 1.10 | 0.532 |  |
| **REMOVAL OF BROILERS (AFV)** | | | | | | | | | | |
| 22 | AFV55 [2] | Transport vehicle cleaned/disinfected before arrival: not always or unknown (ref: always) | AFV | 0.21 | 0.01 | 0.75 | -0.69 | 2.46 | 0.578 |  |
| 23 | AFV58 [2] | Clean/disinfect loading area after each removal: no (ref: yes) | AFV | 0.40 | 0.06 | 0.81 | -0.37 | 2.79 | 0.691 |  |
| 24 | AFV59 [2] | Emptying house in multiple steps: two or more steps (ref: all at once) | AFV | -0.36 | -0.03 | 0.98 | -3.32 | 0.69 | 0.613 |  |
| **REMOVAL OF MANURE AND CARCASSES (MEST)** | | | | | | | | | | |
| 25 | MEST70 [2] | Manure stored on farm: yes (ref: no) | MEST | 0.02 | 0.00 | 0.47 | -0.99 | 1.12 | 0.518 |  |
| 26 | MEST75 [3] | Carcass storage secured against pests: not closed (ref: fully closed) | MEST | 0.09 | 0.00 | 0.72 | -1.15 | 1.95 | 0.532 | Category [2] (partially closed) had no respondents in the data, so it does not appear in the model; categories [3] and [4] were retained as separate levels |
| 27 | MEST75 [4] | No separate carcass storage: not applicable (ref: fully closed) | MEST | -0.13 | -0.01 | 0.69 | -2.06 | 0.87 | 0.549 |  |
| 28 | MEST76 [2] | Clean & disinfect carcass storage after each collection: sometimes or only after production round (ref: always) | MEST | **-2.38** | **-2.31** | 1.79 | -6.28 | **0.06** | 0.932 | Shown in Figure 3; second largest absolute posterior mean coefficient among categorical variables |
| 29 | MEST76 [3] | Clean & disinfect carcass storage after each collection: never (ref: always) | MEST | -0.07 | -0.00 | 0.52 | -1.44 | 0.85 | 0.536 |  |
| 30 | MEST76 [4] | Clean & disinfect carcass storage: no separate carcass storage, not applicable (ref: always) | MEST | 0.14 | 0.01 | 0.61 | -0.69 | 1.83 | 0.574 |  |
| 31 | MEST73MEST80 [2] | Dedicated storage present, but rendering company must enter premises (ref: full access without entry) | MEST | 0.14 | 0.01 | 0.69 | -0.86 | 2.09 | 0.555 | COMBINED variable (MEST73 + MEST80) |
| 32 | MEST73MEST80 [3] | No dedicated storage, but rendering company can collect without entering (ref: full access without entry) | MEST | 0.10 | 0.00 | 0.78 | -1.28 | 2.13 | 0.535 | COMBINED variable (MEST73 + MEST80) |
| 33 | MEST73MEST80 [4] | No dedicated carcass storage AND rendering company must enter farm premises (ref: dedicated storage AND collection without entry) | MEST | -0.27 | -0.02 | 0.98 | -3.24 | 0.76 | 0.575 | COMBINED variable (MEST73 + MEST80) |
| **DISEASE MANAGEMENT (ZIEK)** | | | | | | | | | | |
| 34 | ZIEK87 [2] | Stocking density: >33 kg/m² (ref: ≤33 kg/m²) | ZIEK | **-3.22** | **-3.26** | 1.89 | -7.05 | **0.02** | 0.963 | Shown in Figure 3; largest absolute posterior mean coefficient among categorical variables |
| 35 | ZIEK88 [2] | Multiple age groups present on farm: yes (ref: no) | ZIEK | -0.05 | -0.00 | 0.58 | -1.42 | 1.05 | 0.521 |  |
| **MEASURES BETWEEN COMPARTMENTS (COMP)** | | | | | | | | | | |
| 36 | COMP90COMP95 COMP96COMP97 [2] | Multi-house hygiene protocols: except hand washing (ref: no multiple houses or all protocols followed) | COMP | 0.43 | 0.05 | 0.94 | -0.40 | 3.29 | 0.666 | COMBINED variable (COMP90+95+96+97) |
| 37 | COMP90COMP95 COMP96COMP97 [3] | Multi-house hygiene protocols: lock present, separation not maintained (ref: no multiple houses or all protocols followed) | COMP | -0.20 | -0.01 | 0.84 | -2.48 | 0.83 | 0.568 | COMBINED variable (COMP90+95+96+97) |
| 38 | COMP90COMP95 COMP96COMP97 [5] | Multi-house hygiene protocols: no hygiene lock (ref: no multiple houses or all protocols followed) | COMP | -0.04 | -0.00 | 0.48 | -1.26 | 0.89 | 0.530 | COMBINED variable (COMP90+95+96+97) |
| 39 | COMP100 [2] | Separate, dedicated equipment per poultry house: no (ref: yes) | COMP | -0.14 | -0.01 | 0.58 | -1.81 | 0.70 | 0.581 |  |
| **CLEANING AND DISINFECTION (RND)** | | | | | | | | | | |
| 40 | RND104 [2] | Disinfect poultry houses each cycle: no (ref: yes) | RND | -0.02 | -0.00 | 0.45 | -1.06 | 0.87 | 0.511 |  |
| 41 | RND108 [2] | Clean & disinfect feeding system each cycle: no (ref: yes) | RND | 0.05 | 0.00 | 0.43 | -0.78 | 1.14 | 0.524 |  |
| **MODEL INTERCEPT AND FIT DIAGNOSTICS** | | | | | | | | | | |
| n/a | (Intercept) | Model intercept: log-odds of extensive system at all reference categories | n/a | 1.07 |  | 2.08 | -3.27 | 5.19 | 0.734 | Not interpreted substantively |
| n/a | mean_PPD | Mean posterior predictive distribution: expected proportion of extensive farms | n/a | 0.20 |  | 0.04 | 0.12 | 0.28 | n/a | Fit diagnostic; reflects 15/78 = 19.2% extensive farms in sample |

Note: Coefficients are on the log-odds scale. Reference categories for categorical variables are the first (lowest-numbered) category, which typically corresponds to the lowest-risk response. CrI = credible interval. Biocheck.UGent category abbreviations: INF = Infrastructure, location & housing; ORG = Farm organization & incoming goods; BEZ = Visitors & personnel; EEND = Purchase of day-old chicks; AFV = Removal of broilers; MEST = Removal of manure & carcasses; ZIEK = Disease management; COMP = Measures between compartments; RND = Cleaning & disinfection.
