# Supplementary material for: Biosecurity implementation in indoor and outdoor-access broiler production systems in the Netherlands
Source: Poult Sci. 2026 Oct 12;105(12):107951. doi: 10.1016/j.psj.2026.107951 (PMC13696177; doi:10.1016/j.psj.2026.107951)
Supplement: Supplementary file 5 [file mmc5.docx]

# Supplementary Material E. Temporal sensitivity analysis

The intensive farm surveys in this study span 2015–2023, while the extensive farms were surveyed during dedicated farm visits (December 2023 to May 2024). To assess whether pooling intensive farm data across this period introduces temporal confounding, three complementary sensitivity analyses were conducted. All three use a single cutpoint of 2022, chosen to separate the bulk of historical intensive farm surveys from the most recent surveys, which are closest in time to the extensive farm data collection.

## E.1 Survey date distribution

Table E.1 presents the year distribution of the 63 intensive farm surveys. Applying the 2022 cutpoint yields a pre-2022 subgroup (n = 47; surveyed 2015–2021) and a 2022+ subgroup (n = 16; surveyed 2022–2023). This same split is used for the descriptive comparison (E.2), the period-as-covariate model (E.3), and the recent-period model (E.4).

***Table E.1. Year distribution of intensive farm surveys.***

| **Survey year** | **No. of intensive farms** | **% of intensive** | **Period** |
| --- | --- | --- | --- |
| 2015 | 10 | 15.9% | Pre-2022 |
| 2016 | 6 | 9.5% | Pre-2022 |
| 2017 | 11 | 17.5% | Pre-2022 |
| 2018 | 13 | 20.6% | Pre-2022 |
| 2019 | 4 | 6.3% | Pre-2022 |
| 2020 | 2 | 3.2% | Pre-2022 |
| 2021 | 1 | 1.6% | Pre-2022 |
| 2022 | 7 | 11.1% | 2022+ |
| 2023 | 9 | 14.3% | 2022+ |
| Total | 63 | 100% | Pre-2022: 47 / 2022+: 16 |

## E.2 Descriptive comparison: pre-2022 vs 2022+ (intensive farms)

To assess whether biosecurity responses shifted over time within intensive farms, mean response values for all 28 HPAI-related variables were compared between the pre-2022 (n = 47) and 2022+ (n = 16) subgroups. Standardized differences were calculated as the 2022-onward mean minus the pre-2022 mean, divided by the pooled standard deviation, and were used descriptively to compare the magnitude of changes between periods; no dichotomous threshold was applied. The largest absolute standardized differences were observed for AFV59 (−1.03), ZIEK87 (−0.65), AFV58 (−0.59), BK2 (+0.58) and AFV55 (+0.50). The AFV55 value reflected a difference of only 1 farm (0 of 47 versus 1 of 16) and was unstable because the pre-2022 group showed no variation. Table E.2 summarizes these variables together with the 3 variables illustrated in Figure 3; of those 3, only ZIEK87 was among the largest standardized differences. Figure E.1 shows the response category distributions for the 3 variables illustrated in Figure 3.

***Table E.2. Descriptive comparison of the 3 variables illustrated in Figure 3 and the 4 further variables with the largest standardized differences, between pre-2022 and 2022+ intensive farms.***

| **Code** | **Variable** | **Pre-2022 mean** | **SD** | **2022+ mean** | **SD** | Standardized difference | **Interpretation** |
| --- | --- | --- | --- | --- | --- | --- | --- |
| MEST76 | Carcass storage cleaning | 1.74 | 0.71 | 2.00 | 0.52 | +0.38 | Similar mean; distribution shifted (Figure E.1) |
| ZIEK87 | Stocking density | 1.79 | 0.41 | 1.50 | 0.52 | −0.65 | Moderate difference (78.7% vs 50.0% at category 2) |
| EEND53 | Chick delivery frequency | 2.55 | 0.77 | 2.44 | 0.51 | −0.16 | Similar mean; distribution shifted (Figure E.1) |
| AFV59 | Emptying house in multiple steps | 1.77 | 0.43 | 1.31 | 0.48 | −1.03 | Largest standardized difference; 36 of 47 versus 5 of 16 farms at category 2 |
| AFV58 | Clean/disinfect loading area after each removal | 1.21 | 0.41 | 1.00 | 0.00 | −0.59 | 10 of 47 versus 0 of 16 farms at category 2 |
| BK2 | Years of experience of main farm manager | 25.04 | 9.55 | 31.00 | 12.16 | +0.58 | Continuous variable; mean years |
| AFV55 | Transport vehicle not always cleaned and disinfected before arrival | 1.00 | 0.00 | 1.06 | 0.25 | +0.50 | 1-farm difference; unstable because the pre-2022 group showed no variation |

Note: Response values are coded on ordinal scales where 1 = highest biosecurity compliance. Higher means indicate lower compliance. Standardized difference = (2022+ mean − pre-2022 mean) / pooled SD. For the ordinal variables, where 1 indicates the highest biosecurity compliance, negative values indicate lower coded responses from 2022 onward. For the continuous variable (BK2, years of experience) the sign indicates the direction of change in the original measurement, so the positive value reflects greater mean experience from 2022 onward. The first 3 rows are the variables illustrated in Figure 3; the remaining rows are the variables with the largest absolute standardized differences. For ZIEK87, the proportion of farms at category 2 declined from 78.7% pre-2022 (37 of 47) to 50.0% in 2022+ (8 of 16), which is relevant to interpreting the period-as-covariate model results in E.3. These means summarize the numerical codes as used in the model, with equal spacing assumed for variables with more than 2 levels; changes should be read against the category distributions rather than as a ranking of compliance, since stocking density and chick delivery frequency describe structural or logistical conditions and MEST76 category 4 denotes the absence of separate carcass storage rather than a cleaning frequency.

*Figure E.1. Response category distributions for the 3 biosecurity variables illustrated in Figure 3 (Carcass storage cleaning, Stocking density, Chick delivery frequency) in pre-2022 (n = 47, orange) and 2022+ (n = 16, green) intensive farms. Mean coded responses changed little for MEST76 and EEND53, but the category distributions shifted in both: for MEST76 the proportion reporting cleaning after each collection fell from 36.2% (17 of 47) to 12.5% (2 of 16), and for EEND53 the proportion at category 2 rose from 10.6% (5 of 47) to 56.3% (9 of 16), with no farm in the 2022+ group reporting fewer than 3 deliveries per year. For ZIEK87 (Stocking density), the proportion of farms at category 2 declined from 78.7% pre-2022 to 50.0% in 2022+, a moderate within-intensive shift relevant to interpreting the results in E.3.*

## E.3 Period-as-covariate model (all farms, n = 78)

To formally test whether survey period influenced the main associations between biosecurity practices and production system, the baseline Bayesian logistic regression model was refitted with a binary period indicator added as an additional covariate (0 = pre-2022, 1 = 2022 onward). The indicator took the value 0 for the 47 intensive farms surveyed before 2022 and the value 1 for the 16 intensive farms surveyed from 2022 onward together with all 15 extensive farms, which were surveyed during dedicated farm visits between December 2023 and May 2024. This formulation tests directly whether the coefficients for the biosecurity variables in the main model change when survey period is accounted for. The baseline model was refitted within the same R session to enable a like-for-like comparison of posterior estimates. Both models used identical data, priors, and MCMC settings as specified in Supplementary Material C. All analyses were rerun using a common fixed seed (20240517) to ensure reproducibility and consistency across the baseline and sensitivity-analysis scripts.

Two outcomes are possible. If the posterior estimates for the biosecurity variables are practically unchanged between the baseline and extended models, and the period coefficient is concentrated near zero, survey period does not meaningfully influence the system-level associations observed in the baseline model. If posterior estimates shift substantially when period is added, this may indicate either temporal confounding or structural correlation between period and production system in the dataset.

***Table E.3. Posterior estimates for the 3 variables illustrated in Figure 3 in the baseline (M0) and extended (M1) models, and for the period covariate in the extended model (all farms, n = 78).***

| **Code** | **Variable** | **Model** | **Posterior mean** | **95% CrI** | **Shift (M1 − M0)** |
| --- | --- | --- | --- | --- | --- |
| MEST76 | Carcass storage cleaning | Baseline (M0) | −2.38 | [−6.28, 0.06] | − |
| MEST76 | Carcass storage cleaning | Extended (M1) | −2.82 | [−6.94, 0.06] | −0.44 |
| ZIEK87 | Stocking density | Baseline (M0) | −3.22 | [−7.05, 0.02] | − |
| ZIEK87 | Stocking density | Extended (M1) | −1.31 | [−5.52, 0.27] | +1.91 |
| EEND53 | Chick delivery frequency | Baseline (M0) | 1.08 | [−0.22, 4.59] | − |
| EEND53 | Chick delivery frequency | Extended (M1) | 0.27 | [−0.71, 2.69] | −0.81 |
| − | *Survey period (2022+ vs pre-2022)* | Extended (M1) | 3.96 | [0.01, 8.03] | n/a |

The 3 variables illustrated in Figure 3 behaved differently in the extended model. Posterior means are reported in Table E.3 and in the forest plot (Figure E.2). Because the model uses a horseshoe prior that shrinks weakly supported coefficients toward zero, the comparison focuses on how much each posterior mean changes between models, read together with its credible interval, rather than on whether an interval crosses zero in either model. The MEST76 (carcass storage cleaning) posterior mean shifted only modestly, from −2.38 to −2.82, preserving both direction and magnitude. This estimate is among the largest of all 28 biosecurity variables and changed little when period was added. The EEND53 (chick delivery frequency) posterior mean shifted from 1.08 to 0.27, with the M1 estimate substantially smaller, indicating a weaker and less stable signal. The ZIEK87 (stocking density) posterior mean, by contrast, shifted substantially, from −3.22 to −1.31, with the period coefficient absorbing much of the between-system variation in stocking density, indicating that the ZIEK87 signal is not stable across model specifications. Across all 28 biosecurity variables in the model, for 30 of the 38 other parameters the absolute shift in the posterior mean was smaller than 0.10, and the largest shift among all 38 parameters was 0.30. Corresponding posterior median shifts were all smaller than 0.10 (maximum 0.09), reflecting asymmetry in some horseshoe posterior distributions, as posterior means are more sensitive than medians to the tails of skewed distributions. The correlation of posterior means between the models was 0.88 after excluding the 3 illustrated categorical variables and 0.86 overall, with the lower overall correlation driven by the ZIEK87 shift.

The period coefficient itself was large (posterior mean 3.96, 95% credible interval [0.01, 8.03]). This should be interpreted with caution because period and production system are structurally correlated in the dataset: all 15 extensive farms were surveyed from 2022 onward, and no pre-2022 extensive farms exist. As a result, the 47 farms with period = 0 are all intensive, while the 31 farms with period = 1 include all extensive farms together with the 16 intensive farms surveyed from 2022 onward. A portion of the period coefficient therefore reflects this structural correlation rather than a purely temporal effect. The ZIEK87 shift reflects a statistical identifiability problem: within intensive farms, pre-2022 surveys recorded more farms at ZIEK87 category 2 (78.7%, n = 37 of 47) than did 2022+ surveys (50.0%, n = 8 of 16), meaning stocking density is correlated with survey period within the intensive subgroup (see Figure E.1). Because all extensive farms are simultaneously 2022+ and at category 1, the model cannot separate the period signal from the stocking density signal, and adding period as a covariate draws variance from ZIEK87. Whether the within-intensive temporal shift in stocking density reflects management changes after 2021, or is an artifact of which farms participated in surveys in different years, cannot be determined from this dataset. Taken together, the MEST76 estimate changed comparatively little when survey period was included, whereas the ZIEK87 estimate attenuated substantially, indicating sensitivity to temporal adjustment and reflecting an identifiability limitation inherent to the study design rather than a methodological issue in the analysis.

Figure E.2. Posterior coefficient summaries for the 3 biosecurity variables illustrated in Figure 3 in the baseline model (M0, green) and the extended model with survey period as an additional covariate (M1, purple). Point = posterior mean. Thick bar = 90% credible interval. Thin bar = 95% credible interval. MEST76 changes modestly between models (−2.38 to −2.82); EEND53 attenuates toward zero (1.08 to 0.27); ZIEK87 attenuates substantially (−3.22 to −1.31) as the period coefficient absorbs the between-system variation in stocking density.

## E.4 Recent-period model (2022–2023 intensive + 2023–2024 extensive, n = 31)

To assess whether the baseline model associations hold when the comparison is restricted to the most recent surveys, a reduced model was fitted on the subset of intensive farms surveyed from 2022 onward (n = 16) combined with all extensive farms surveyed during dedicated farm visits (n = 15, 2023–2024), totaling 31 observations. This restriction compares the two production systems within the most recent survey period, reducing the possibility that observed associations are artifacts of comparing recent extensive data with older intensive data.

Given the limited sample size, the full 28-variable model could not be supported; instead, a focused model was fitted with only the 3 variables illustrated in Figure 3 (MEST76, ZIEK87, EEND53) and two covariates (BK2, BK3). A regularized horseshoe prior with slightly weaker global shrinkage was used to accommodate the smaller sample (global_scale = 0.15 instead of 0.075; df = global_df = 1, slab_df = 4, slab_scale = 2.5; sampling settings as in Supplementary Material C). All other MCMC settings were identical to the baseline model. Table E.4 and the accompanying text report posterior means, consistent with the convention used throughout this supplementary material. This model differs from the baseline in sample, adjustment set and prior scale as well as in survey period, so it tests a combined alternative specification rather than survey period alone.

***Table E.4. Posterior estimates for the 3 variables illustrated in Figure 3 in the recent-period model (n = 31).***

| **Code** | **Variable** | **Mean** | **95% CrI lower** | **95% CrI upper** | **Interpretation** |
| --- | --- | --- | --- | --- | --- |
| MEST76 | Carcass storage cleaning | −4.10 | −8.82 | −0.16 | Large negative estimate; direction retained in the recent-period model, which also differed in sample, adjustment set and prior scale |
| ZIEK87 | Stocking density | −1.47 | −7.24 | 0.37 | Attenuated toward zero; consistent with period-adjusted estimate |
| EEND53 | Chick delivery frequency | n/a | n/a | n/a | Baseline contrast unavailable; category 1 absent in this subset |

MEST76 (carcass storage cleaning) produced a large negative posterior mean in the recent-period model (posterior mean −4.10, 95% CrI [−8.82, −0.16]), comparable in magnitude to the baseline model estimate (posterior mean −2.38, 95% CrI [−6.28, 0.06]) and slightly larger. The estimated direction remained negative in the smaller recent-period model. Because this analysis also changed the sample, adjustment set and prior scale, it cannot isolate the effect of survey period. ZIEK87 (stocking density) was attenuated toward zero, consistent with the period-adjusted estimate in M1 and with reduced variation in the smaller sample. EEND53 (chick delivery frequency) is reported as n/a in Table E.4 because no farm in this subset reported fewer than 3 deliveries per year, so the category 2 versus category 1 contrast shown for the other models does not exist here; the variable remained in the model with category 2 as the reference, and the category 3 versus category 2 contrast had a posterior mean of 0.08 (95% CrI [−1.43, 2.19]).

## E.5 Summary

Across the specifications examined, the MEST76 (carcass storage cleaning) estimate was the most consistent, whereas the ZIEK87 (stocking density) estimate was sensitive to the inclusion of survey period and the EEND53 (chick delivery frequency) estimate attenuated after period adjustment. Descriptive comparisons show small differences in mean coded response for MEST76 and EEND53 and a moderate difference for ZIEK87, although the category distributions of all 3 shifted to some degree (E.2). The period-as-covariate model shows that the MEST76 posterior mean is stable across M0 and M1 (posterior means −2.38 and −2.82 respectively, shift of only 0.44 on the log-odds scale), while the ZIEK87 estimate attenuates substantially (shift of 1.91), reflecting a statistical identifiability problem inherent to the study design rather than a methodological artifact (E.3). In the recent-period model the MEST76 estimate remained negative and comparable in magnitude (posterior mean −4.10) when the comparison was restricted to records from 2022 onward (E.4). Taken together, the sensitivity analyses suggest that the MEST76 estimate was more consistent across specifications than the ZIEK87 and EEND53 estimates, but they do not establish that it is free of temporal confounding.

These results are consistent with a farm-level difference in biosecurity practices between production systems, although residual temporal confounding cannot be excluded. The ZIEK87 association remains informative as a descriptive feature of extensive versus intensive systems but is less cleanly separable from survey period within this dataset, a limitation we acknowledge in the Discussion.
