# Supplementary material for: Biosecurity implementation in indoor and outdoor-access broiler production systems in the Netherlands
Source: Poult Sci. 2026 Oct 12;105(12):107951. doi: 10.1016/j.psj.2026.107951 (PMC13696177; doi:10.1016/j.psj.2026.107951)
Supplement: Supplementary file 6 [file mmc6.docx]

**Supplementary Material F. Prior and model sensitivity analyses**

To assess the robustness of the baseline model findings to analytical choices, two sensitivity analyses were conducted: (SA1) varying the horseshoe prior’s global shrinkage parameter, and (SA2) excluding structurally system-linked variables from the model. Sensitivity was examined within the regularized horseshoe family, which is suited to the sparse modeling setting of this study; alternative prior families were not assessed. All analyses used the same dataset (n = 78; intensive = 63, extensive = 15) and MCMC settings as the baseline model (4 chains, 4,000 iterations, adapt_delta = 0.9999; see Supplementary Material C) unless otherwise noted.

**F.1 Prior sensitivity: horseshoe global_scale (SA1)**

The horseshoe prior’s global_scale parameter controls the overall degree of shrinkage; smaller values impose stronger shrinkage toward zero and larger values impose weaker shrinkage. In the baseline model, global_scale was set to 0.075, which encodes a prior expectation that most coefficients are near zero. To test whether the associations for the 3 variables illustrated in Figure 3 are sensitive to this choice, the model was refitted with global_scale values of 0.05 (stronger shrinkage, expecting fewer signals) and 0.15 (weaker shrinkage, expecting more signals).

*Table F.1. Posterior estimates for the 3 variables illustrated in Figure 3 across horseshoe global_scale values.*

| **Variable** | **global_scale** | **Mean** | **95% CrI lower** | **95% CrI upper** | **90% CrI lower** | **90% CrI upper** | **P(\|coef\| > 0.1)** |
| --- | --- | --- | --- | --- | --- | --- | --- |
| **Carcass storage cleaning [2]** | 0.05 | −2.33 | −6.19 | 0.06 | −5.48 | 0.02 | 0.87 |
|  | 0.075 | −2.38 | −6.28 | 0.06 | −5.50 | 0.01 | 0.89 |
|  | 0.15 | −2.40 | −6.40 | 0.10 | −5.65 | 0.02 | 0.90 |
| **Stocking density [2]** | 0.05 | −3.18 | −7.10 | 0.03 | −6.32 | −0.01 | 0.93 |
|  | 0.075 | −3.22 | −7.05 | 0.02 | −6.36 | −0.02 | 0.94 |
|  | 0.15 | −3.13 | −6.99 | 0.03 | −6.25 | −0.03 | 0.95 |
| **Chick delivery frequency [2]** | 0.05 | 1.02 | −0.20 | 4.37 | −0.11 | 3.73 | 0.66 |
|  | 0.075 | 1.08 | −0.22 | 4.59 | −0.12 | 3.89 | 0.69 |
|  | 0.15 | 1.25 | −0.23 | 4.73 | −0.12 | 4.11 | 0.76 |

Posterior means for MEST76 (carcass storage cleaning) ranged from −2.33 to −2.40 across the three global_scale values, a shift of 0.07 across a three-fold range of shrinkage intensity. ZIEK87 (stocking density) was similarly stable, with means between −3.13 and −3.22. EEND53 (chick delivery frequency) showed a modest increase in mean from 1.02 (global_scale = 0.05) to 1.25 (global_scale = 0.15), consistent with the weaker shrinkage allowing more of its signal through, but the direction and overall pattern were unchanged. The estimates were similar across the three global_scale settings examined.

*Figure F.1. Posterior mean and credible intervals for the 3 variables illustrated in Figure 3 across horseshoe global_scale values (SA1). Thick bars = 90% CrI; thin bars = 95% CrI; dots = posterior mean. MEST76 and ZIEK87 retain consistent negative associations across all three shrinkage levels. EEND53 shows a modest increase in posterior mean with weaker shrinkage but maintains the same direction.*

**F.2 Reduced model excluding structurally system-linked variables (SA2)**

Two of the 3 variables illustrated in Figure 3, EEND53 (chick delivery frequency) and ZIEK87 (stocking density), are inherently linked to the intensive/extensive distinction. Stocking density is constrained by regulatory maximum densities that differ between production systems, and chick delivery frequency is determined by the number of production cycles, which is structurally lower in extensive systems with longer growing periods. These variables therefore partly define the production system rather than representing independent biosecurity choices.

To examine how the MEST76 estimate changed when these structurally system-linked variables were removed, the baseline model was refitted with 26 variables (excluding EEND53 and ZIEK87). All other model settings were identical to the baseline.

*Table F.2. MEST76 (carcass storage cleaning) posterior estimates: baseline vs reduced model.*

| **Model** | **Mean** | **95% CrI lower** | **95% CrI upper** | **Change** |
| --- | --- | --- | --- | --- |
| Baseline (28 variables) | −2.38 | −6.28 | 0.06 | - |
| Reduced (26 variables, excl. EEND53 + ZIEK87) | −2.14 | −6.05 | 0.09 | ~10% |

The MEST76 coefficient changed modestly between the full and reduced models: the posterior mean shifted from −2.38 to −2.14, a change of about 10%, and the 95% credible interval remained similar [−6.05, 0.09]. This supports the stability of the MEST76 estimate when these two predictors are excluded, although residual confounding cannot be excluded.

In the reduced model, the coefficient for emptying the house in multiple steps (AFV59 [2]) changed from −0.36 in the baseline model to −3.06 (95% CrI −6.64 to 0.00). This category occurred among 41 intensive farms and no extensive farms, producing a strongly separated response pattern. Its increased coefficient after removing chick delivery frequency and stocking density is consistent with redistribution of system-separating information among related variables and should not be interpreted as a newly identified independent association. Apart from this change, the horseshoe prior continued to shrink most coefficients toward zero, and the overall sparsity pattern was preserved. Approximate PSIS-LOO favored the full model: the reduced model had an elpd_loo 4.9 points lower than the full model (SE 1.6). PSIS-LOO was computed using the relative efficiency of the MCMC draws, as in Supplementary Material C. One observation in each model had a Pareto k value marginally above 0.7 (0.704 in the full model and 0.775 in the reduced model), and exact leave-one-out refitting of those 2 observations left the difference unchanged. The full model was therefore retained as the primary analysis.

*Figure F.2. Forest plot for the reduced model (26 variables, excluding EEND53 and ZIEK87). Dashed vertical line at zero. Dark bar = 90% CrI; light bar = 95% CrI; dot = posterior mean. MEST76 [2] (carcass storage cleaning) retains a negative posterior mean comparable to the baseline model. Emptying house in multiple steps (AFV59 [2]) increases substantially in this model, consistent with redistribution of system-separating information among related variables.*

**F.3 Summary**

The prior and model sensitivity analyses produced broadly similar estimates across the specifications examined. MEST76 (carcass storage cleaning) and ZIEK87 (stocking density) had stable negative posterior means across the three levels of horseshoe shrinkage (SA1). MEST76 also retained a similar estimate when EEND53 and ZIEK87 were excluded (SA2). EEND53 (chick delivery frequency) showed a modest increase in posterior mean under weaker horseshoe shrinkage (global_scale = 0.15 vs 0.05), consistent with the horseshoe appropriately treating it as a less clearly separable signal.

These analyses indicate limited sensitivity of the MEST76 estimate to the prior scales and predictor sets examined. Its estimated direction also remained negative in the recent-period analysis in Supplementary Material E, although residual temporal confounding cannot be excluded.
