# Supplementary material for: Biosecurity implementation in indoor and outdoor-access broiler production systems in the Netherlands
Source: Poult Sci. 2026 Oct 12;105(12):107951. doi: 10.1016/j.psj.2026.107951 (PMC13696177; doi:10.1016/j.psj.2026.107951)
Supplement: Supplementary file 7 [file mmc7.docx]

**Supplementary Material G. STROBE checklist for cross-sectional studies**

*Manuscript:* Biosecurity implementation in indoor and outdoor-access broiler production systems in the Netherlands

**Legend:** ✓ = Addressed	|	N/A = Not applicable

| **Item No** | **STROBE Recommendation** | **Reported on Page/Section** | **Status** |
| --- | --- | --- | --- |
| ***Title and Abstract*** | | | |
| **1a** | Indicate the study's design with a commonly used term in the title or the abstract | Abstract: "A cross-sectional comparative study was conducted to assess HPAI-related biosecurity practices...". | ✓ |
| **1b** | Provide in the abstract an informative and balanced summary of what was done and what was found | Abstract: states the design ("A cross-sectional comparative study"), what was done (78 farms; 63 intensive and 15 extensive; harmonized Biocheck.UGent subset; 40 comparable variables, 28 selected; Bayesian logistic regression with horseshoe priors), and what was found (most biosecurity measures did not differ substantially between systems, with considerable variation within both systems; the largest estimated differences involved carcass storage cleaning, stocking density and chick delivery frequency, with varying statistical support). | ✓ |
| ***Introduction*** | | | |
| **2** | Explain the scientific background and rationale for the investigation being reported | Introduction: scientific background (HPAI threat and Dutch context, biosecurity as the primary control, intensive versus extensive systems and their differing exposure pathways, knowledge gap on production-system comparisons) and rationale for a within-regulatory-context, HPAI-relevant comparison. | ✓ |
| **3** | State specific objectives, including any prespecified hypotheses | Introduction, final paragraph: states the aim ("this study compared HPAI-related biosecurity practices between intensive and extensive Dutch broiler farms using a harmonized subset of Biocheck.UGent questions aligned with recognized pathways of HPAI introduction and spread") and the prespecified hypothesis ("We hypothesized that although system-level structural characteristics would differ, substantial variation in biosecurity practices would remain between farms within each system"). | ✓ |
| ***Methods*** | | | |
| **4** | Present key elements of study design early in the paper | Materials and Methods, Study Design and Farm Selection (first sentence): "A cross-sectional study was conducted to compare biosecurity practices and risk factors between intensive and extensive broiler farms in the Netherlands." | ✓ |
| **5** | Describe the setting, locations, and relevant dates, including periods of data collection | Materials and Methods, Biosecurity Assessment and Data Collection: setting is the Netherlands; extensive farms visited in person between December 7, 2023 and May 2, 2024; intensive surveys obtained from the Biocheck.UGent database (survey years 2015-2023). | ✓ |
| **6a** | Give the eligibility criteria, and the sources and methods of selection of participants | Materials and Methods, Study Design and Farm Selection: production systems defined by outdoor access (indoor-only = intensive; covered outdoor access = extensive); transitional-certification and niche systems (e.g., Kipster) excluded; intensive farms drawn from the Biocheck.UGent database; extensive farms recruited through professional networks (veterinary contacts and industry associations). | ✓ |
| **7** | Clearly define all outcomes, exposures, predictors, potential confounders, and effect modifiers | Materials and Methods, Statistical Analysis: outcome is production system (binary; intensive = 0, extensive = 1); the 28 selected biosecurity variables are entered as independent variables (full list in Supplementary Material A); residual confounding is acknowledged in the Discussion (Strengths and Limitations). | ✓ |
| **8*** | For each variable of interest, give sources of data and details of methods of assessment. Describe comparability of assessment methods if there is more than one group | Materials and Methods, Biosecurity Assessment and Data Collection: both groups assessed with the Biocheck.UGent scoring system using two survey versions (extensive, 100 questions; intensive, 79 questions); extensive surveys completed on-site by researchers or veterinarians, intensive surveys obtained from the database. Comparability procedures are described under Data Cleaning and Harmonization (Supplementary Material A). | ✓ |
| **9** | Describe any efforts to address potential sources of bias | Materials and Methods: on-site administration of extensive surveys reduced reporting bias; assessors agreed beforehand on the interpretation of the questions, and visits not conducted by the main assessors were documented with photographs and written descriptions; harmonization performed independently by two researchers with consensus; question-exclusion criteria (low variability, at least 14 of 15 extensive and 59 of 63 intensive farms in one category, in both systems; high missingness) were predefined a priori, before examining system-level differences, to avoid data-driven selection. | ✓ |
| **10** | Explain how the study size was arrived at | Materials and Methods, Study Design and Farm Selection: no formal sample size calculation (the required effect size and variability were not available at design); extensive group = all eligible consenting farms (n = 15); intensive group = all Dutch broiler surveys available in the Biocheck.UGent database at extraction (17 May 2024; n = 63, approximately 10% of the 613 Dutch broiler farms in 2023). | ✓ |
| **11** | Explain how quantitative variables were handled in the analyses | Materials and Methods, Statistical Analysis: continuous variables (years of experience of the main farm manager, number of persons employed) modeled as continuous; categorical variables modeled as unordered factors with dummy coding (category 1 as reference); low-count categories merged before modeling (Supplementary Material A). For visualization, risk scores were min-max normalized to a 0-1 range. | ✓ |
| **12a** | Describe all statistical methods, including those used to control for confounding | Materials and Methods, Statistical Analysis: Bayesian logistic regression with a regularized horseshoe prior (rstanarm v2.32.2; R v4.5.2); 4 chains of 4,000 iterations (2,000 warmup) giving 8,000 posterior samples; adapt_delta = 0.9999; convergence assessed with R-hat (all = 1.00) and effective sample size; fit assessed with posterior predictive checks and PSIS-LOO cross-validation (loo v2.9.0) with Pareto k diagnostics and an approximate PSIS-LOO cross-validated AUC as an additional model check (Supplementary Material C); associations summarized using posterior means, 95% credible intervals and posterior probabilities of direction (Supplementary Material D). | ✓ |
| **12b** | Describe any methods used to examine subgroups and interactions | Not applicable: no subgroup or interaction analyses in the main model. | ✓ N/A |
| **12c** | Explain how missing data were addressed | Materials and Methods, Data Cleaning and Harmonization: one variable excluded for 52.4% missingness in the intensive dataset; "No other missing data were present." | ✓ |
| **12d** | If applicable, describe analytical methods taking account of sampling strategy | Materials and Methods and Discussion: intensive farms from a database and extensive farms purposively recruited (non-random sampling); potential selection bias and representativeness limits discussed in Strengths and Limitations. | ✓ |
| **12e** | Describe any sensitivity analyses | Materials and Methods, Statistical Analysis: three temporal sensitivity analyses using a 2022 cut point (pre-2022 versus from-2022 distribution comparison; full model with survey period as a covariate; recent-period model on the overlapping subsample, n = 31), plus prior and model sensitivity analyses (Supplementary Materials E and F). | ✓ |
| ***Results*** | | | |
| **13a** | Report numbers of individuals at each stage of study | Results (first sentence): "Biosecurity was compared across 78 farms (63 intensive, 15 extensive)." | ✓ |
| **13b** | Give reasons for non-participation at each stage | Materials and Methods, Study Design and Farm Selection: the total number of farmers approached was not recorded, precluding a participation rate; a small number of farms were excluded post-consent under the predefined criteria; intensive farms came from a pre-existing database. Potential selection bias discussed in Strengths and Limitations. | ✓ |
| **13c** | Consider use of a flow diagram | Not included (a flow diagram is optional for cross-sectional studies). | ✓ N/A |
| **14a** | Give characteristics of study participants and information on exposures and potential confounders | Results and Supplementary Material B: full descriptive statistics (frequencies and percentages) for all 40 harmonized variables stratified by production system; the 3 variables with the largest estimated contrasts are described in the Results text and Figure 3. | ✓ |
| **14b** | Indicate number of participants with missing data for each variable of interest | Materials and Methods, Data Cleaning and Harmonization: one variable excluded for 52.4% missingness (intensive dataset); no other missing data for retained variables. | ✓ |
| **15*** | Report numbers of outcome events or summary measures | Results: intensive n = 63 (coded 0) and extensive n = 15 (coded 1); response-category counts and percentages reported for the 3 variables shown in Figure 3 (all variables in Supplementary Material B). | ✓ |
| **16a** | Give unadjusted estimates and, if applicable, confounder-adjusted estimates and their precision | Results: posterior means with 95% credible intervals and posterior probabilities of direction for the 3 categorical variables with the largest absolute coefficients: carcass storage cleaning "Sometimes" -2.38 (95% CrI -6.28 to 0.06; 0.93), stocking density above 33 kg/m2 -3.22 (95% CrI -7.05 to 0.02; 0.96), chick delivery "3-6 times" +1.08 (95% CrI -0.22 to 4.59; 0.80); full coefficient output in Supplementary Material D. | ✓ |
| **16b** | Report category boundaries when continuous variables were categorized | Materials and Methods and Results: stocking density threshold 33 kg/m2; chick delivery categories (fewer than 3, 3-6, more than 6 times per year). | ✓ |
| **16c** | If relevant, consider translating estimates of relative risk into absolute risk | Not applicable: coefficients are reported on the log-odds scale; the study is a cross-sectional comparison rather than a risk-prediction model. | ✓ N/A |
| **17** | Report other analyses done (e.g., subgroup and interaction analyses, and sensitivity analyses) | Results and Supplementary Materials E and F: temporal sensitivity analyses (distribution comparison, survey-period covariate model, overlapping-subsample model) and prior/model sensitivity analyses; summarized in Results and Discussion. | ✓ |
| ***Discussion*** | | | |
| **18** | Summarise key results with reference to study objectives | Discussion (first paragraph): with reference to the study aim, summarizes the limited evidence for systematic differences between production systems, the substantial variation within both systems, and the 3 largest estimated differences with varying statistical support; within- and between-system variance components were not formally estimated. | ✓ |
| **19** | Discuss limitations of the study, taking into account sources of potential bias or imprecision. Discuss both direction and magnitude of any potential bias | Discussion, Strengths and Limitations: measurement error from self-reported items (may overestimate compliance) and differential framing of 2 component questions (Supplementary Material A); non-random sampling and a convenience intensive sample (possible bias toward higher biosecurity awareness); unknown participation rate; differences in assessment mode, for which the direction and magnitude of bias cannot be determined; limited representativeness (no flock size; province known for 16 of 63 surveys); temporal comparability (Supplementary Material E); limited information per coefficient (n = 15 extensive farms); residual confounding; exclusion of system-specific questions; no linkage to HPAI incidence. Where the direction of a bias could not be determined, this is stated. | ✓ |
| **20** | Give a cautious overall interpretation of results | Discussion: cautious interpretation throughout (e.g., estimates near zero "should be read as inconclusive rather than as evidence of similarity"; the stocking density association "should be interpreted cautiously"), integrating limitations, prior literature, and alternative explanations. | ✓ |
| **21** | Discuss the generalisability (external validity) of the study results | Discussion: external validity addressed by conducting the comparison within a single regulatory context and by explicit caveats on the representativeness of the intensive convenience sample and its limited geographic information; the Conclusions call for validation in larger and longitudinal studies that incorporate system-specific exposure pathways. | ✓ |
| ***Other Information*** | | | |
| **22** | Give the source of funding and the role of the funders | The Funding section: funded by the European Union’s Horizon Europe research and innovation program under Grant Agreement No. 101083923 (BIOSECURE Enhanced and cost-effective biosecurity in livestock production). | ✓ |
